# Supplementary material for: Multi-omic features of oesophageal adenocarcinoma in patients treated with preoperative neoadjuvant therapy
Source: Nat Commun. 2023 May 31;14:3155. doi: 10.1038/s41467-023-38891-x (PMC10232490; doi:10.1038/s41467-023-38891-x)
Supplement: Supplementary file 1 — Supplementary Information File [file 41467_2023_38891_MOESM1_ESM.docx]

**SUPPLEMENTARY INFORMATION­**

Multi-omic features of oesophageal adenocarcinoma in patients treated with preoperative neoadjuvant therapy, Naeini *et al*

­
Supplementary Figure 1: Overview of mutations in known oesophageal adenocarcinoma driver genes.
Supplementary Figure 2: Rearrangement signatures and HRD scores present in oesophageal adenocarcinoma.

Supplementary Figure 3: Association with stage and cox regression analysis.

Supplementary Figure 4: APOBEC mutational signature in TCGA cohort.

Supplementary Figure 5: Overview of copy number alteration events in known oesophageal cancer driver genes.

Supplementary Figure 6: Ploidy and overall stage in samples without complex events.

Supplementary Figure 7: Overview of sub-clonal CNA percentage across genomes.

Supplementary Figure 8: Complex genomes and kataegis.

Supplementary Figure 9: Examples of chromosomes harbouring the most frequent APOBEC kataegic loci and maximum percentage of overlap with rearrangement breakpoints.

Supplementary Figure 10: Methylation status of *EGFR* promoter and gene-body.

Supplementary Figure 11: Methylation status of *MTMR9* promoter and gene-body.

Supplementary Figure 12: Methylation status of *GATA4* promoter and gene-body.

Supplementary Figure 13: Methylation status of *GATA6* promoter and gene-body.

Supplementary Figure 14: Methylation status of *SMAD4* promoter and gene-body.

Supplementary Figure 15: Optimal number of clusters for k-mean clustering in our study cohort and TCGA cohort.

Supplementary Figure 16: Pearson correlation between CIBERSORTx and MethylCIBERSORT.

Supplementary Figure 17: Immune microenvironment of TCGA cohort.

Supplementary Figure 18: Gene set enrichment analysis of Cluster 3 and Cluster 4.

Supplementary Figure 19: Heterogeneity of CD8 immunohistochemistry in tumours with immune hot microenvironment (Cluster 1).

Supplementary Figure 20: Heterogeneity of CD8 immunohistochemistry in tumours with immune suppressed microenvironment (Cluster 2).

Supplementary Figure 21: Heterogeneity of CD8 immunohistochemistry in tumours with immune moderate microenvironment (Cluster 3).

Supplementary Figure 22: Heterogeneity of CD8 immunohistochemistry in tumours with immune cold microenvironment (Cluster 4).

Supplementary Figure 23: Clinico-pathological features of samples in each immune cluster.

Supplementary Figure 24: Genomic features associated with the immune clusters.

Supplementary Figure 25: Immune checkpoint molecules associated with the immune clusters.

­

**
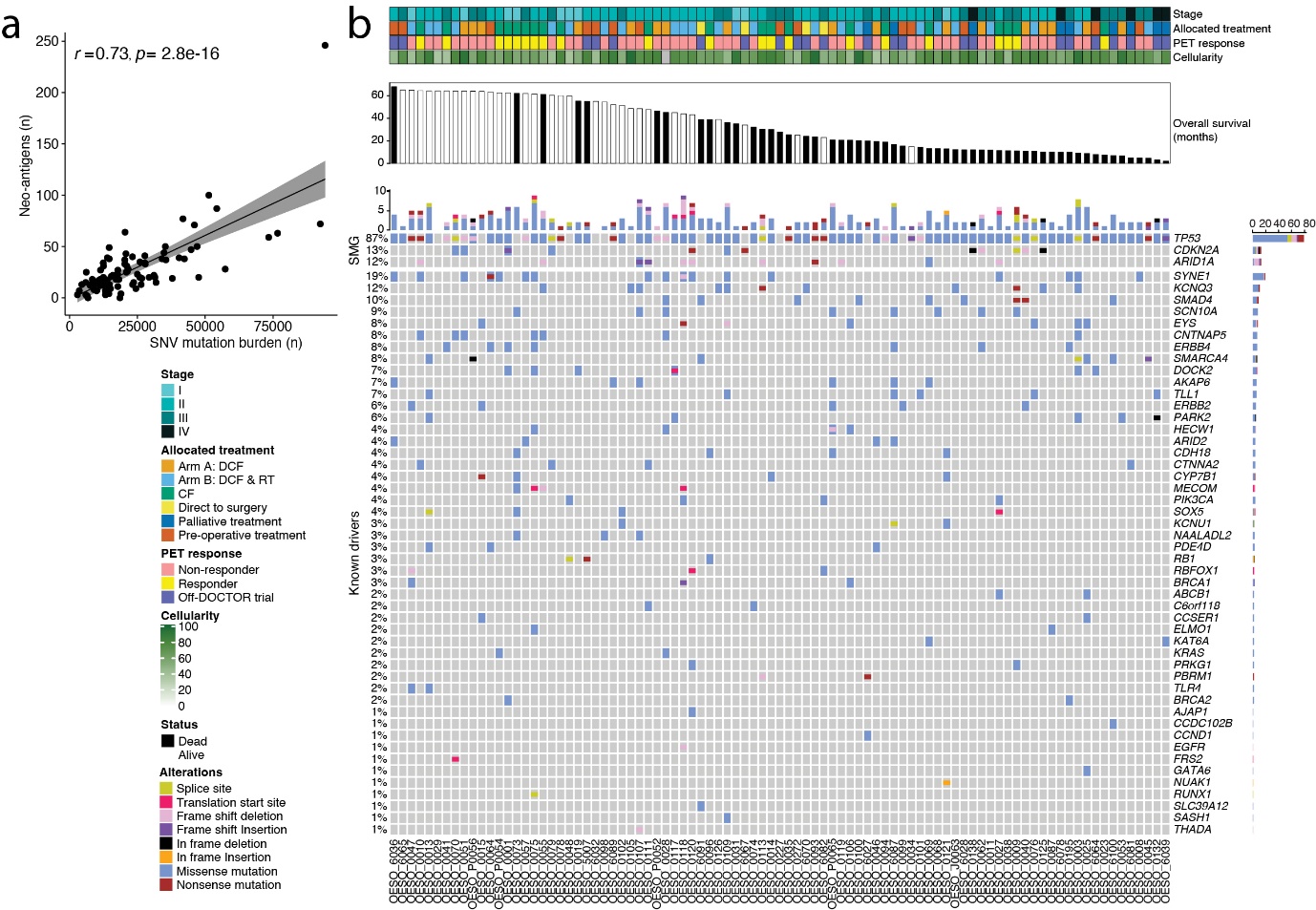
**

**Supplementary Figure 1: Overview of mutations in known oesophageal adenocarcinoma driver genes.** a) Pearson correlation between the number of predicted neoantigens with the number of SNVs per sample (n = 89 biologically independent samples). Shading indicates 95% confidence intervals. b) The colour bar above the figure represents from top to bottom: overall stage, allocated treatment, PET response and tumour cellularity. The histogram is the overall patient survival in months (y-axis), white bars represents patients who are alive and black who are dead. The next histogram is the total number of mutations in driver genes for each sample (n = 89 biologically independent samples). The oncoplot shows the mutations in driver genes that are significantly mutated in this cohort (*TP53*, *CDKN2A* and *ARID1A*) and mutations in previously reported driver genes according to published articles. SMG, significantly mutated gene; PET, Positron Emission Tomography; CF, Cisplatin and 5-Fluorouracil; DCF, CF and docetaxel; RT, 45Gy radiotherapy. Source data are provided as a Source Data file.


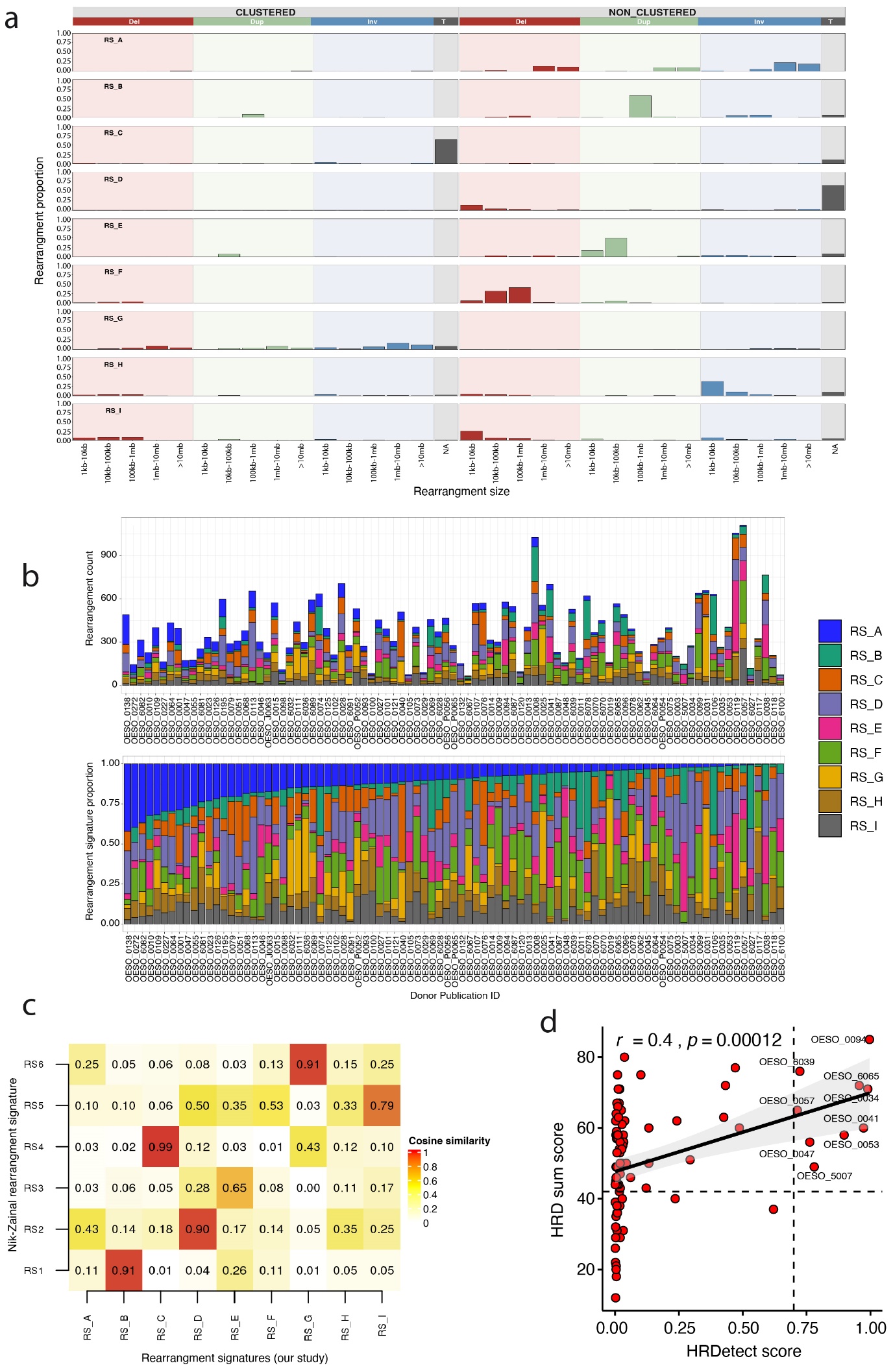
­

**Supplementary Figure 2:** **Rearrangement signatures and HRD scores present in oesophageal adenocarcinoma.** a) Nine rearrangement signatures (RSA-I) were extracted from n = 89 OAC samples using non-negative matrix factorization (NMF) and categorised by clustered and non-clustered deletions (Del), duplications (Dup), Inversions (Inv) and translocations (T). The category type is shown on the x-axis and distinct sizes on the y-axis. b) The contribution of rearrangement signatures are shown for each sample (n = 89 biologically independent samples) in the number of events (upper panel) and proportion of rearrangement signatures (lower panel). c) Cosine similarity of rearrangement signatures detected (x-axis) with published signatures (Nik-Zainal et al., 2016) (y-axis) classified the SV signatures as RS1-6-like. d) Pearson correlation (two-sided) of the homologous recombination deficiency (HRD) score using HRDsum (y-axis) and HRDetect score (x-axis) estimated in OAC samples (n = 89 biologically independent samples). Shading indicates 95% confidence intervals. Dashed lines indicate thresholds for what is considered as HRD by each tool: HRDsum score > 42 and HRDetect > 0.7. Source data are provided as a Source Data file.


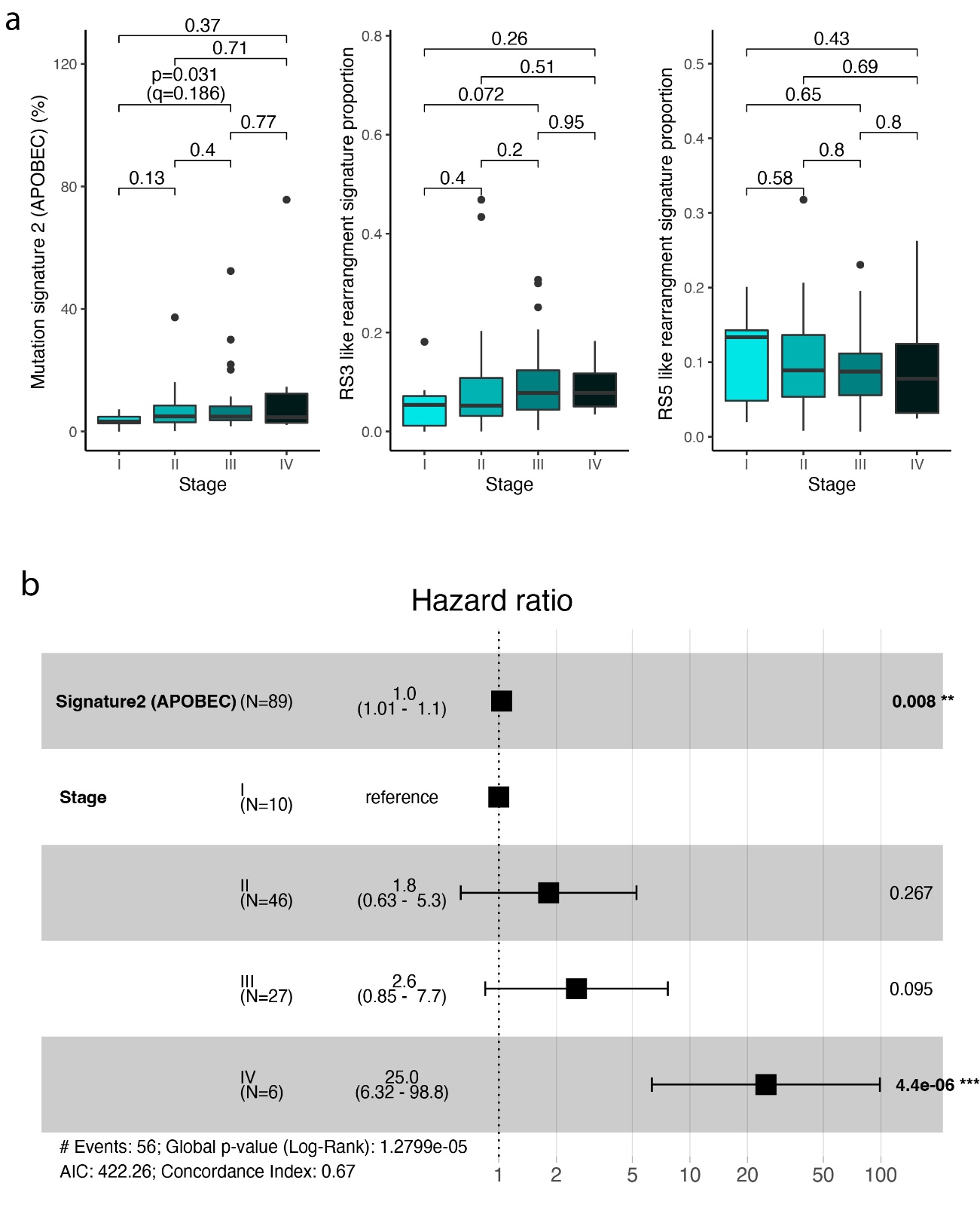


**Supplementary Figure 3:** **Association of mutational signatures with stage and cox regression analysis.** a) Box plots of the SNV mutation signature 2 associated with APOBEC, the rearrangement signatures RS3-like and RS5-like in different tumour stages (stage I n = 10, stage II n = 46, stage III n = 27 and stage IV n = 6, all representing biologically independent samples). p-values from Wilcoxon rank sum two-sided test. Box plots show the median values with the interquartile range (lower and upper hinge) and ± 1.5-fold the interquartile range from the first and third quartile (lower and upper whiskers). b) Cox regression analysis of overall survival and the SNV mutation signature 2 (APOBEC) adjusting for stage in OAC samples (n = 89 biologically independent samples). Error bars are mean values ± SEM. Source data are provided as a Source Data file.


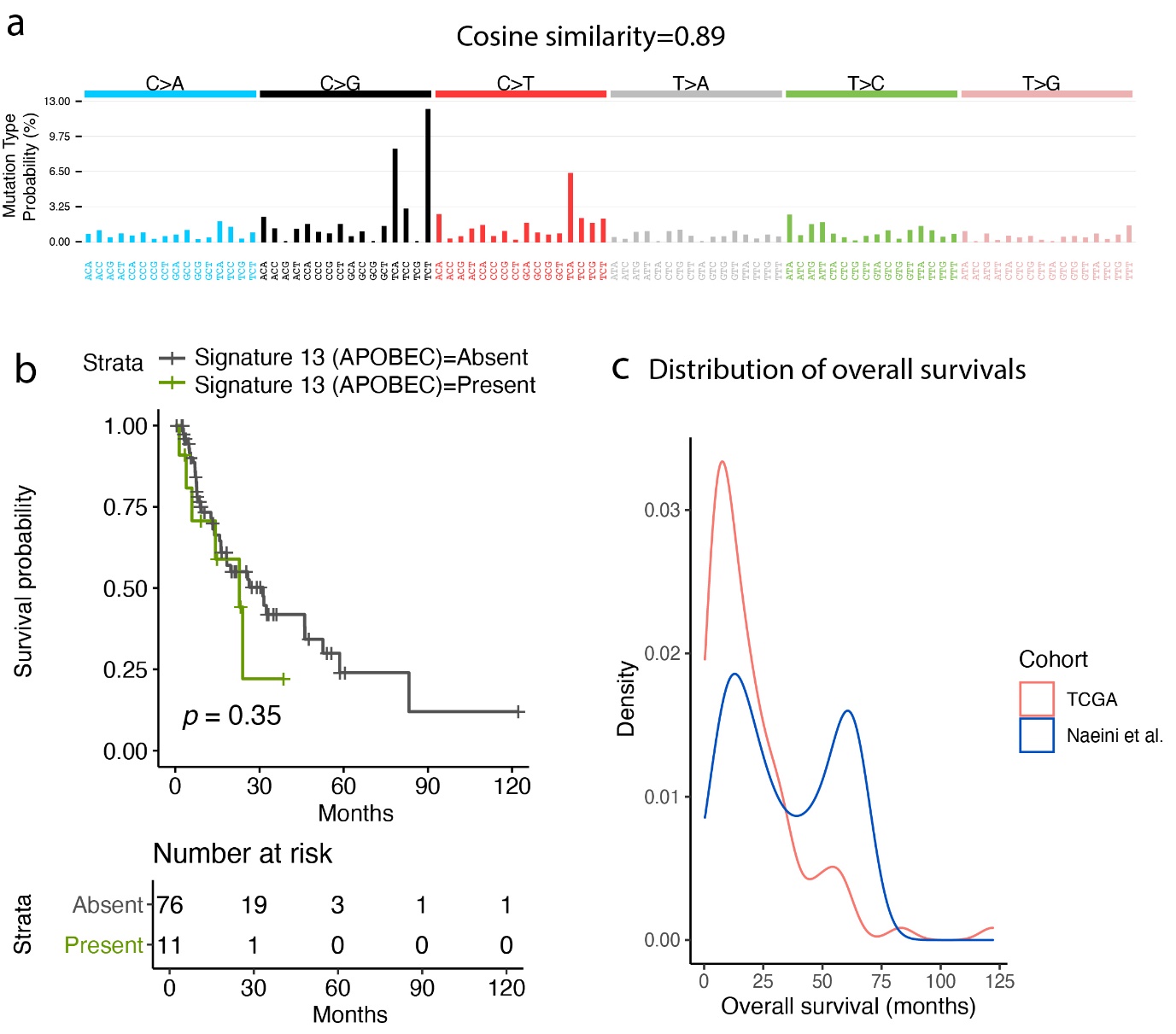


**Supplementary Figure 4: APOBEC mutational signature in TCGA cohort.** a) The APOBEC mutational signature (signature 13) was detected from SNV data of TCGA OAC samples (n = 87) with a cosine similarity of 0.89 compared to COSMIC v2 mutational signatures (https://cancer.sanger.ac.uk/cosmic/signatures_v2). b) Kaplan-Meier plot (log-rank test) comparing overall survival of samples with the APOBEC mutational present (≥ 15% in a sample, n = 11 samples) and absent (< 15%, n = 76 samples). c) Density plot comparing the distribution of overall survival in Naeini et al. (n = 89) and TCGA (n = 87) cohorts. Source data are provided as a Source Data file.

­­


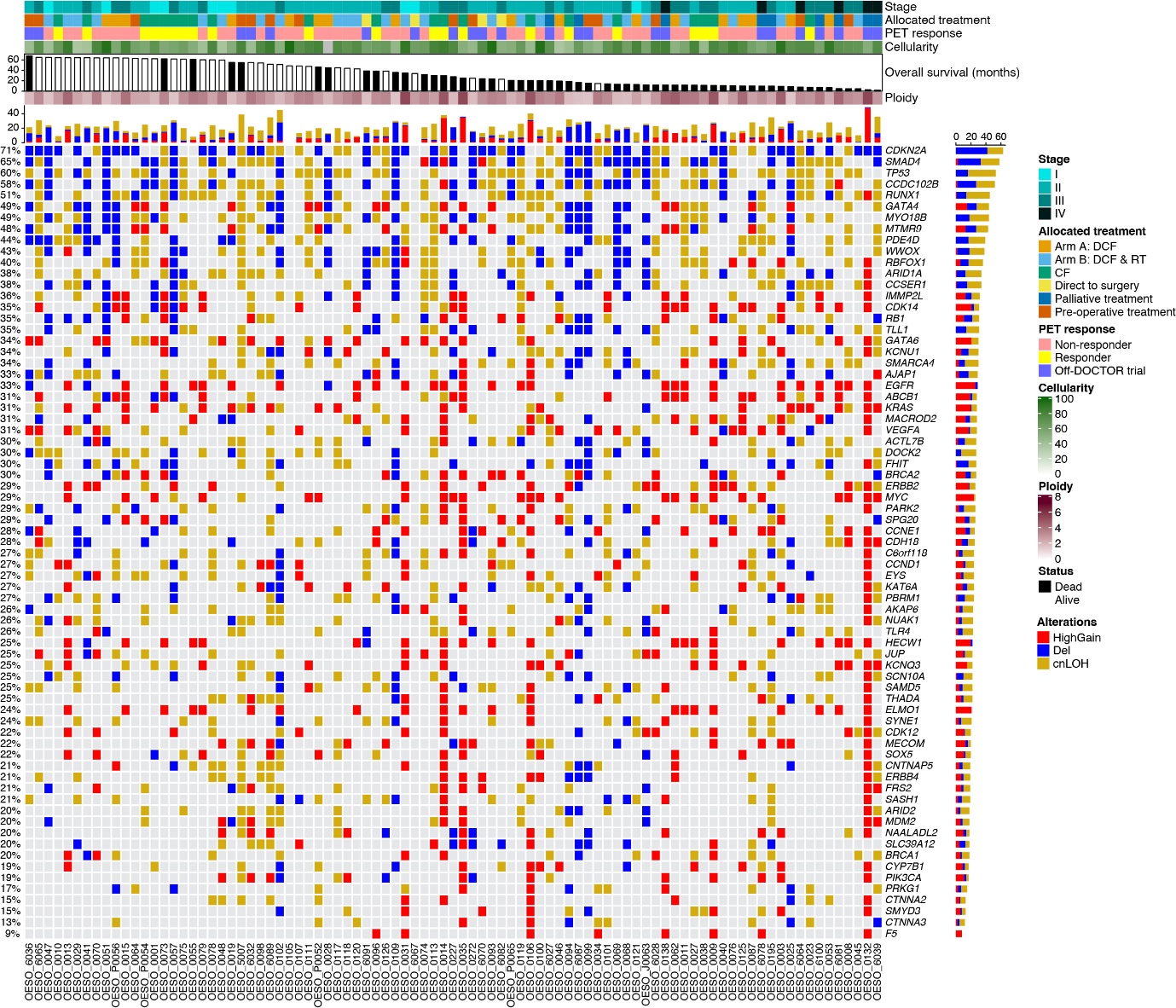


**Supplementary Figure 5:** **Overview of copy number alteration events in known oesophageal cancer driver genes.** Copy number data for n = 89 biologically independent samples. The colour bar above the figure represents from top to bottom: overall stage, allocated treatment, PET response and tumour cellularity. The histogram is the overall patient survival (months), white bars are patients who are alive and black who are dead. The ploidy is shown for each sample. Copy number (CN) shown for known oesophageal driver genes (y-axis) for each sample (x-axis) and is coloured by HighGain (CN ≥ 6, red), Del (CN0 and CN1, blue) and copy number neutral Loss of heterozygosity (cnLOH, yellow). Right panel: total number of copy number alterations for each driver gene. PET, Positron Emission Tomography; CF, Cisplatin and 5-Fluorouracil; DCF, CF and docetaxel; RT, 45Gy radiotherapy. Source data are provided as a Source Data file.


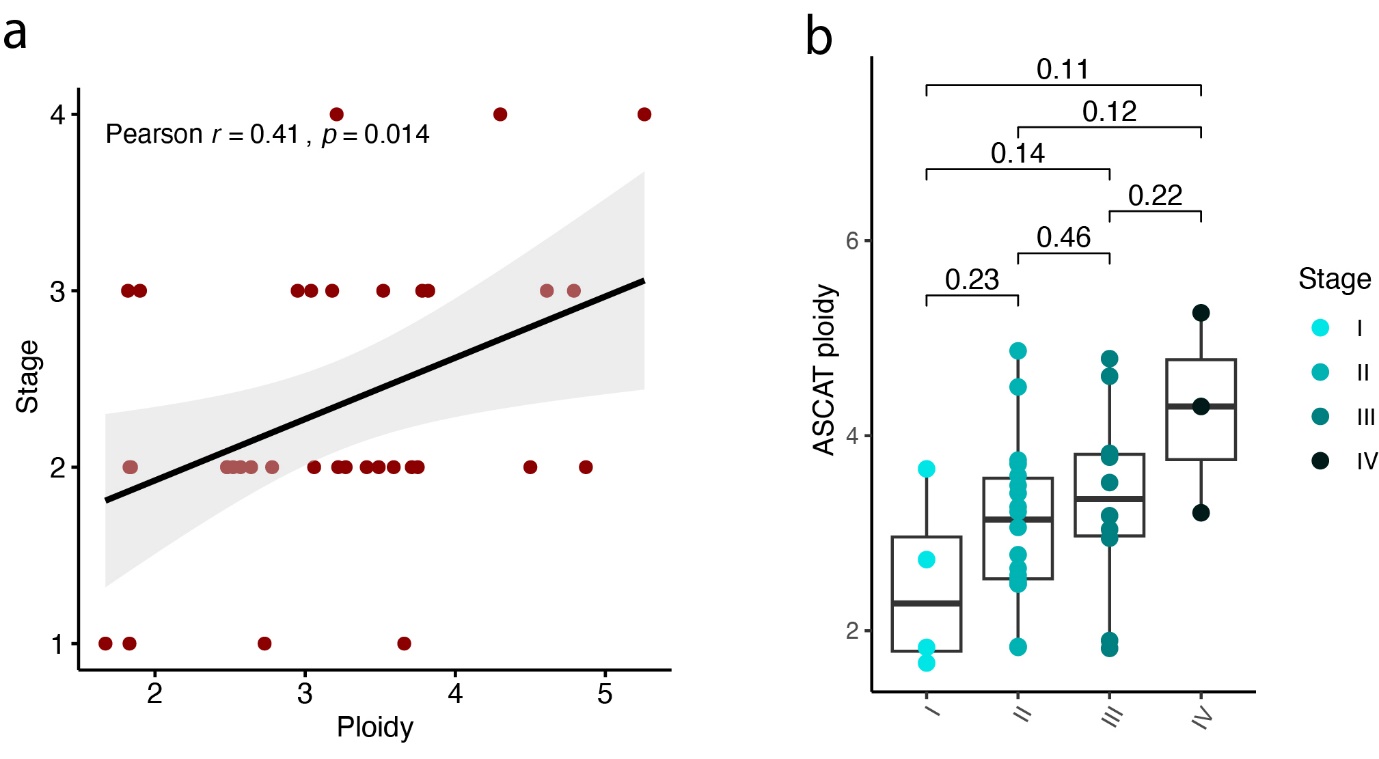


**Supplementary Figure 6: Ploidy and overall stage in samples without complex events.** a) Pearson correlation (two-sided) of the overall stage of tumour (I, II, III and IV) (y-axis) compared to ploidy determined by ASCAT (x-axis) for each sample without complex events (n = 35 biologically independent samples). Shading indicates 95% confidence intervals. b) Box plots of ASCAT ploidy (y-axis) in samples without complex events grouped by tumour stage (x-axis, Stage I n=4, stage II n=18, stage III n=10, stage IV n=3) with p-values determined from Wilcoxon rank sum test. Box plots show the median values with the interquartile range (lower and upper hinge) and ±1.5-fold the interquartile range from the first and third quartile (lower and upper whiskers). Source data are provided as a Source Data file.

­
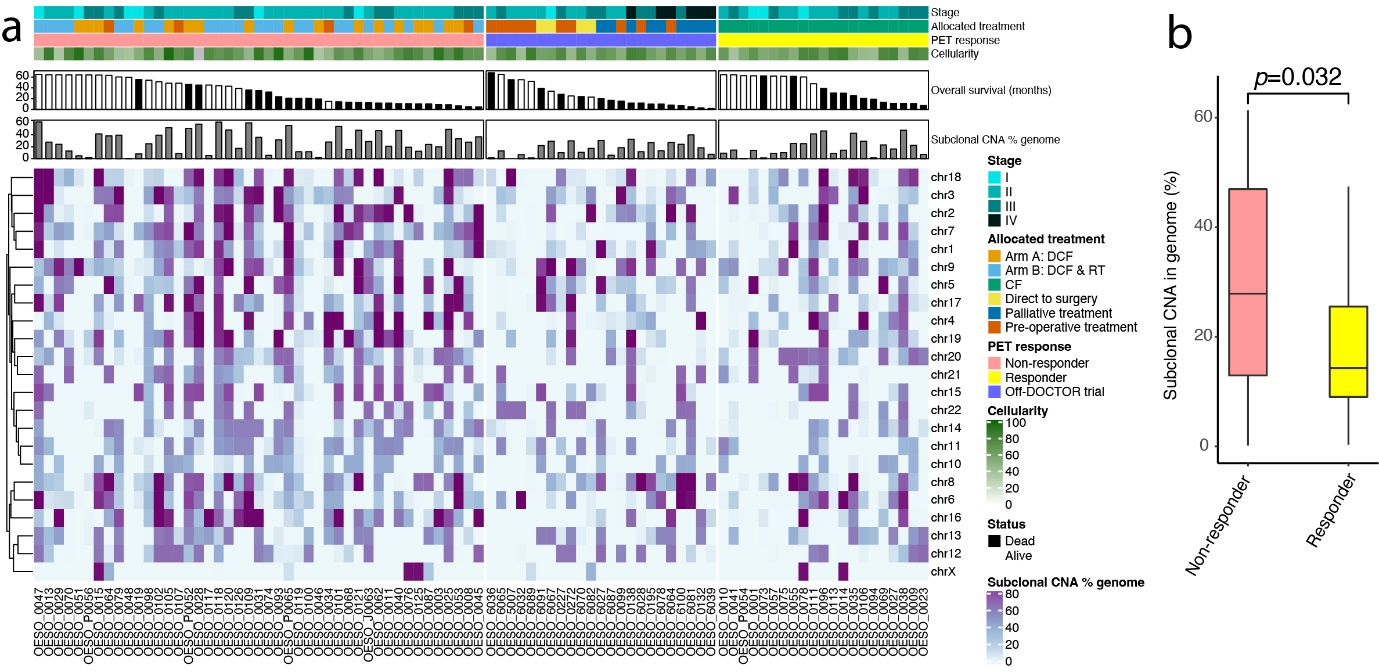
­

**Supplementary Figure 7:** **Overview of sub-clonal CNA percentage across genomes.** a) The colour bar above the figure represents from top to bottom: the overall stage, allocated treatment, PET response and tumour cellularity. The histogram is the overall patient survival (months), white bars are patients who are alive and black who are dead. The samples are sorted by PET response, then overall survival. The next histogram is the percent of the genome affected by sub-clonal copy number aberrations (CNA) in each sample, with hierarchical clustering of sub-clonal CNA percentage identified for each chromosome shown. Samples are named on the x-axis (n = 89 biologically independent samples). b) Box plot of Sub-clonal CNA percentage identified in the genome (Wilcoxon rank sum two-sided test) for patients stratified into PET responders (n = 21) and non-responders (n = 45). Box plot shows the median values with the interquartile range (lower and upper hinge) and ±1.5-fold the interquartile range from the first and third quartile (lower and upper whiskers). PET, Positron Emission Tomography; CF, Cisplatin and 5-Fluorouracil; DCF, CF and docetaxel; RT, 45Gy radiotherapy; CNA, copy number aberrations. Source data are provided as a Source Data file.


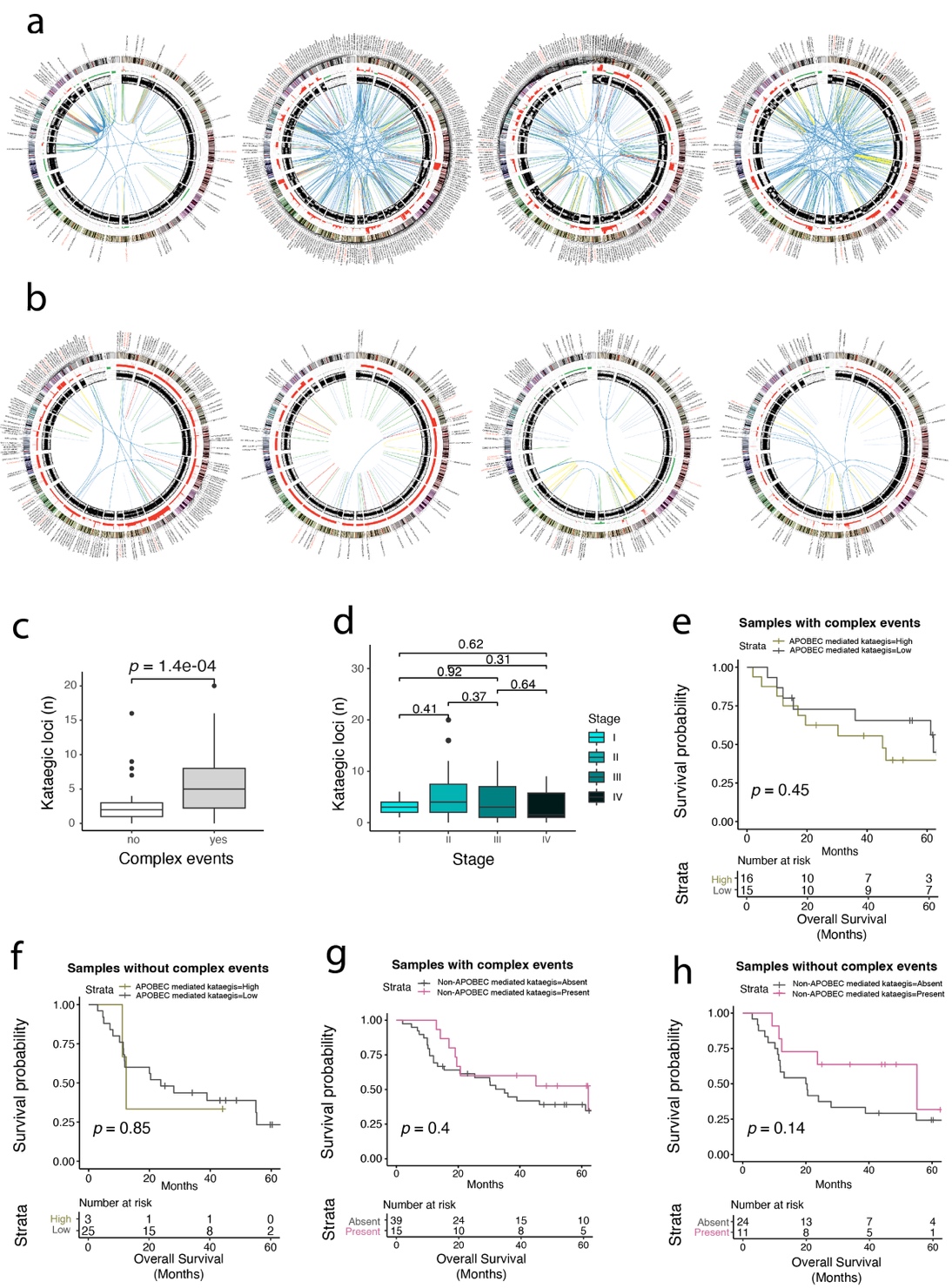
­

**Supplementary Figure 8: Complex genomes and kataegis.** a) Circos plots showing four representative tumours with complex genomes (from left to right samples are: OESO_0040, OESO_0008, OESO_6039 and OESO_0113). Source data for all samples (not just the representative samples) is included in the Source Data file. b) Circos plots showing four representative tumours without complex genomes (from left to right: OESO_0120, OESO_0048, OESO_0019, OESO_0105). For circos plots, the rings show from outer to inner: Chromosome banding, copy number alterations (green is loss and red is gain), BAF and somatic structural variants. Different structural variants are coloured. c) Box plot of the number of kataegic loci in tumors without (n = 35) and with (n = 54) complex genomes with p-values from Wilcoxon rank sum two-sided test. d) Box plots of kataegic loci in samples with different stages (stage I n = 10, stage II n = 46, stage III n = 27 and stage IV n = 6, all representing biologically independent samples) with p-values from Wilcoxon rank sum two-sided test. Box plots in c and d show the median values with the interquartile range (lower and upper hinge) and ± 1.5-fold the interquartile range from the first and third quartile (lower and upper whiskers). p-values from Wilcoxon rank sum two-sided test. e) Kaplan-Meier plot for overall survival (log-rank test) for samples with complex genome events stratified by APOBEC mediated kataegis high (kataegis >=7, n = 16 samples) and low (kataegis <= 2, n = 15 samples) groups. f) Kaplan-Meier plots for overall survival (log-rank test) for samples without complex genome stratified by APOBEC mediated kataegis high (kataegis >= 7, n = 3) and low (kataegis <= 2, n = 25) groups. g) Kaplan-Meier (log-rank test) for samples with complex genome events stratified by non-APOBEC-mediated kataegis present (kataegis >=1, n = 15 samples) and absent (kataegis = 0, n = 39 samples) groups. h) Kaplan-Meier (log-rank test) for samples without complex genome events stratified by non-APOBEC mediated kataegis present (kataegis >=1, n = 11 samples) and absent (kataegis = 0, n = 24 samples) groups. Source data are provided as a Source Data file.

**
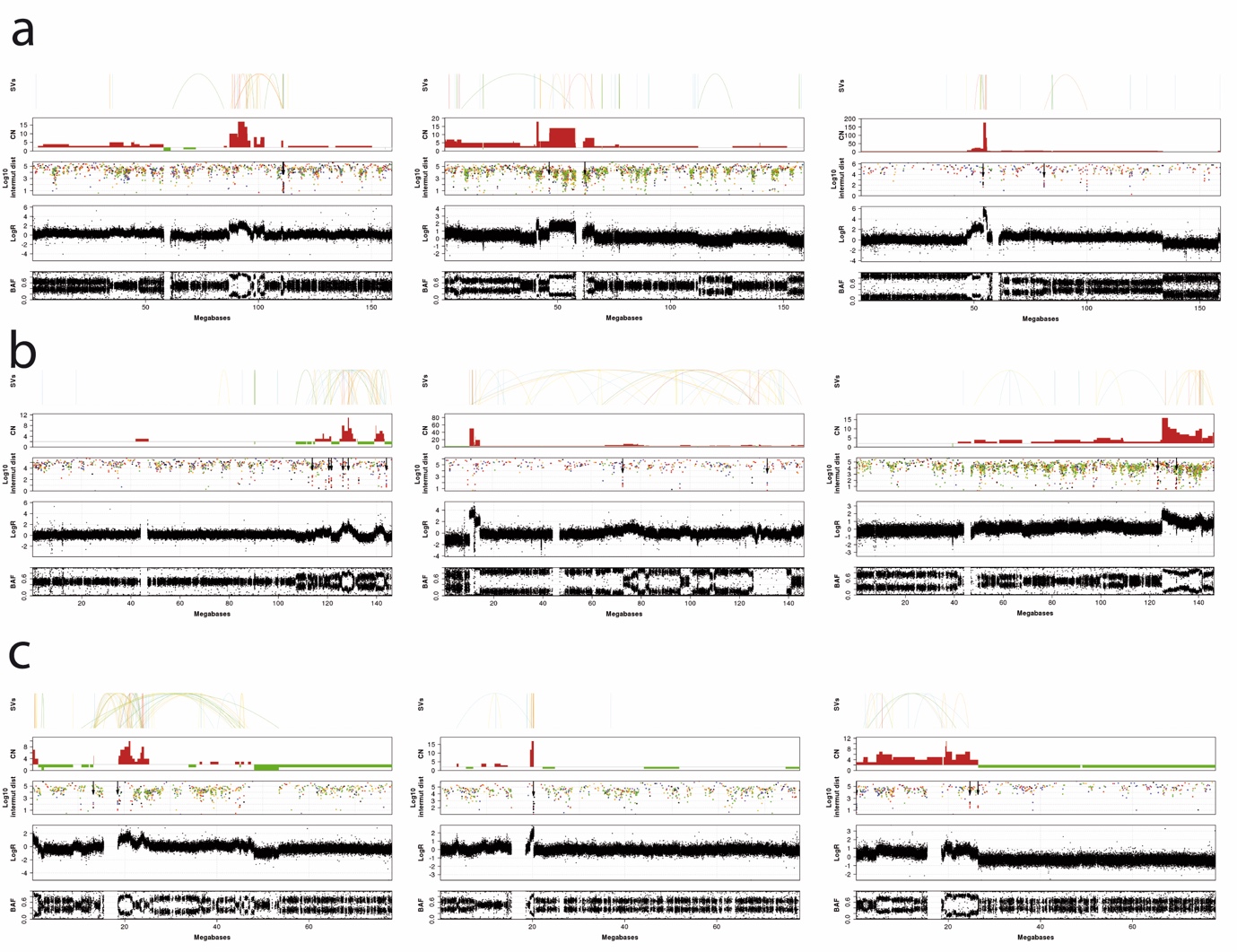
**

**Supplementary Figure 9:** **Examples of chromosomes harbouring the most frequent APOBEC kataegic loci and maximum percentage of overlap with rearrangement breakpoints.** Three representative OAC samples (left to right) are plotted for the three chromosomes: a) chromosome 7; b) chromosome 8 and c) chromosome 18. In each panel, the plots show from upper to lower: density of breakpoints, copy number aberrations (red is gain and green is loss), rainfall plots of SNVs, the logR representing copy number across the chromosome and the BAF. Within the rainfall plots, the black arrows indicate kataegic localised hypermutations.


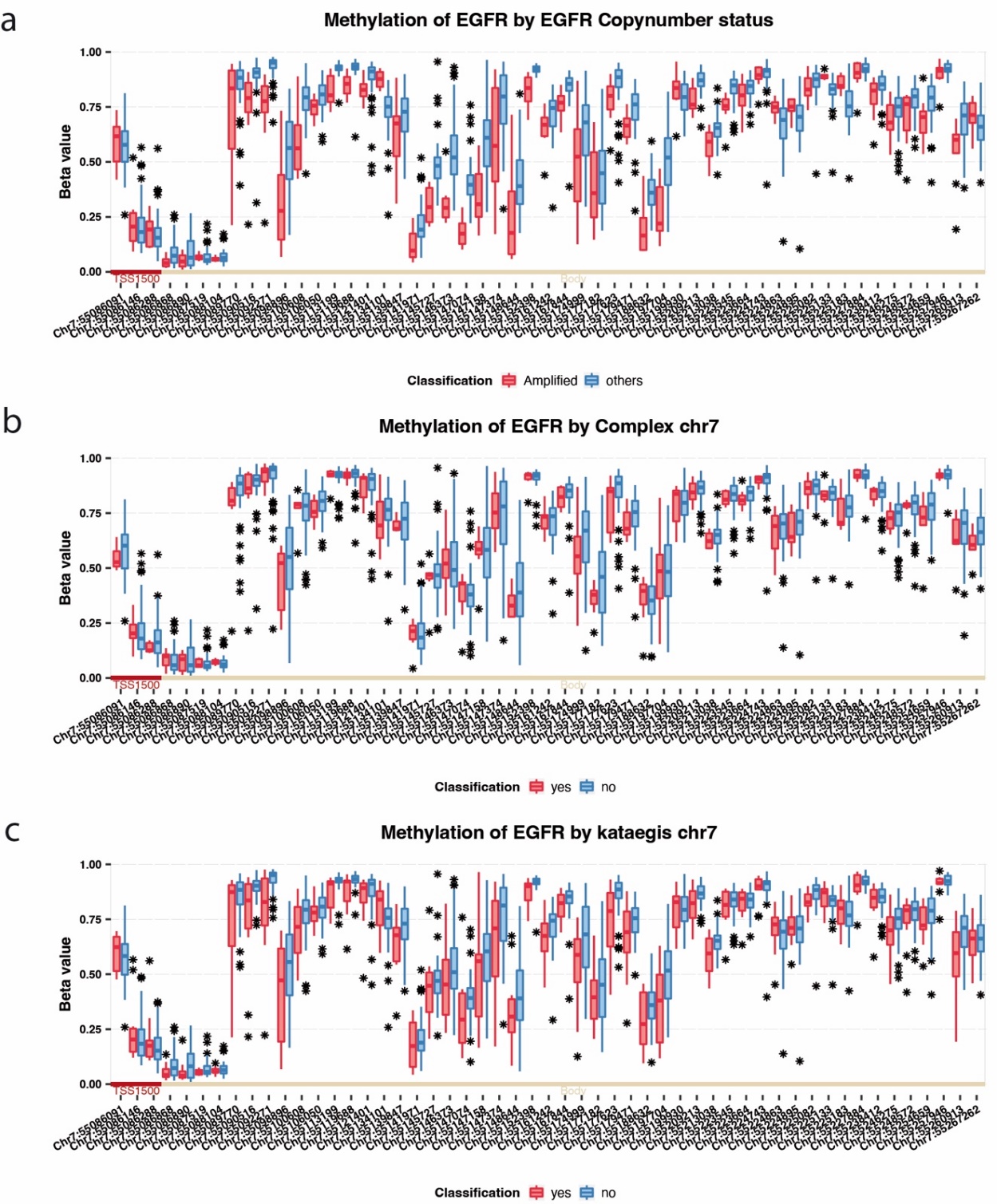


**Supplementary Figure 10: Methylation status of *EGFR* promoter and gene-body**. Data in this figure is derived from n = 69 biologically independent samples. a) Boxplots of beta values for samples with amplified *EGFR* copy number status (n = 8, red) and others (n = 61, blue). Amplified status of the gene of interest has been corrected for ploidy, meaning that samples are considered to have amplification of the gene if ASCATploidy is < 2.7 and the gene copy number is > 5 or where ASCATpoidy is >=2.7 and the gene copy number is >=9. b) Boxplots of beta values for samples with complex chromosome 7 (n = 7, red) and others (n = 62, blue). c) Boxplots of beta values for samples with kataegic events at chromosome 7 (n = 11, red) and others (n = 58, blue). All box plots show the median values with the interquartile range (lower and upper hinge) and ± 1.5-fold the interquartile range from the first and third quartile (lower and upper whiskers), extreme outliers are shown as asterisks ‘*’. Source data are provided as a Source Data file.

_
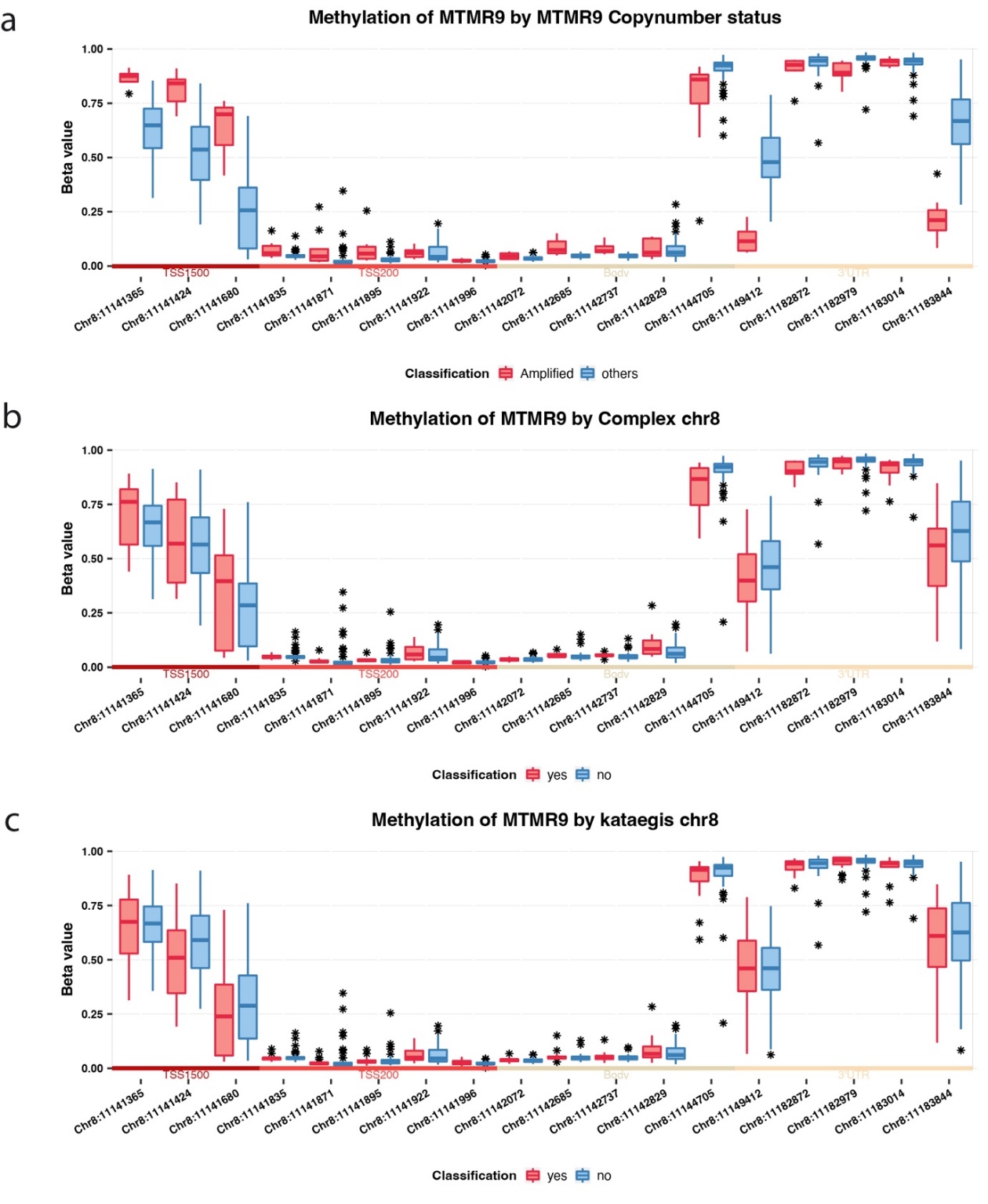
_

**Supplementary Figure 11: Methylation status of *MTMR9* promoter and gene-body**. Data in this figure is derived from n = 69 biologically independent samples. a) Boxplots of beta values in samples with amplified *MTMR9* copy number status (n = 8, red) and others (n = 61, blue). Amplified status of the gene of interest has been corrected for ploidy, meaning that samples are considered to have amplification of the gene if ASCATploidy is < 2.7 and the gene copy number is > 5 or where ASCATpoidy is >=2.7 and the gene copy number is >=9. b) Boxplots of beta values for samples with complex chromosome 8 (n = 8, red) and others (n = 61, blue). c) Boxplots of beta values for samples with kataegic events at chromosome 8 (n = 18, red) and others (n = 51, blue). All box plots show the median values with the interquartile range (lower and upper hinge) and ± 1.5-fold the interquartile range from the first and third quartile (lower and upper whiskers), extreme outliers are shown as asterisks ‘*’. Source data are provided as a Source Data file.

_
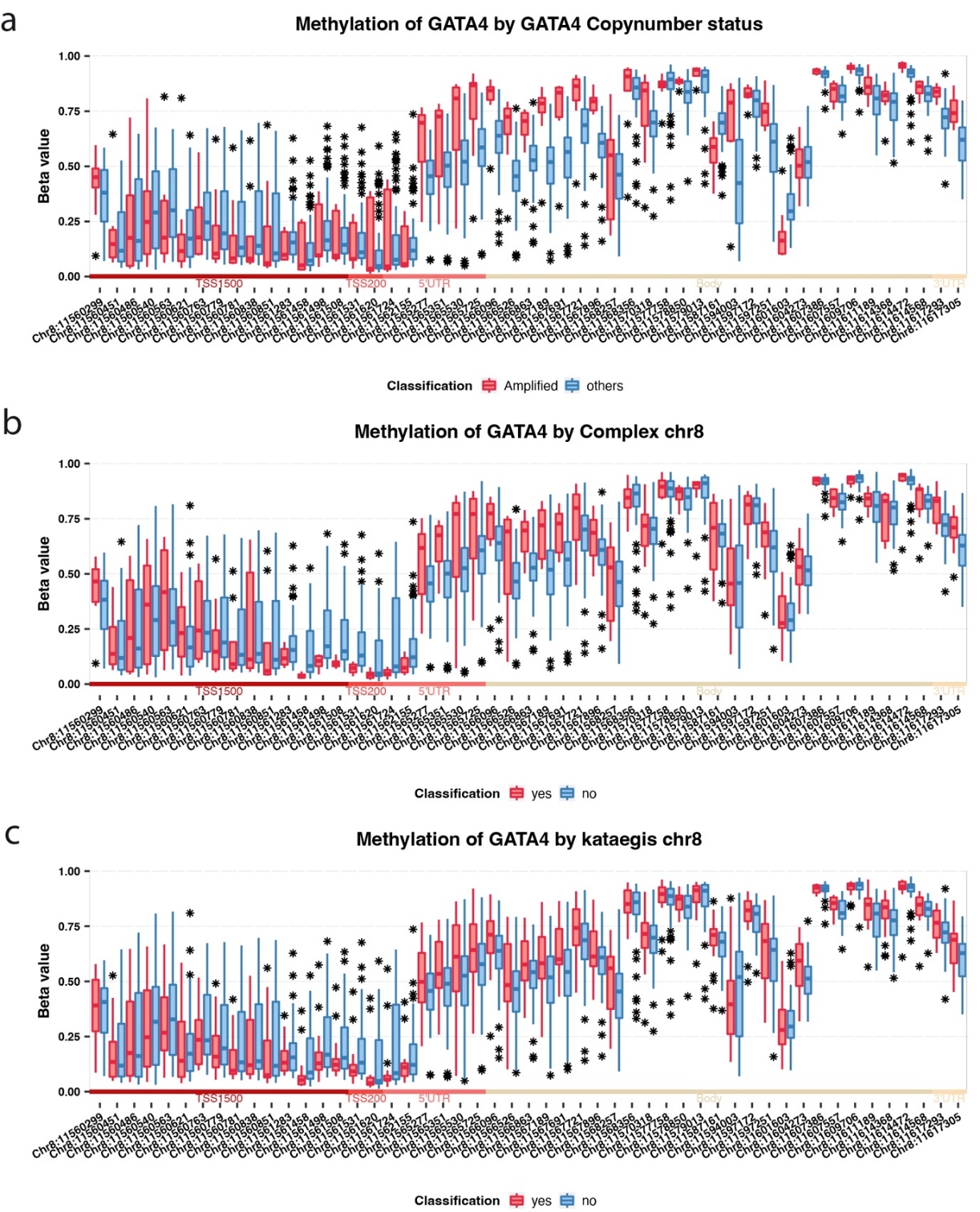
_

**Supplementary Figure 12: Methylation status of *GATA4* promoter and gene-body**. Data in this figure is derived from n = 69 biologically independent samples. a) Boxplots of beta values in samples with amplified *GATA4* copy number status (n = 8, red) and others (n = 61, blue). Amplified status of the gene of interest has been corrected for ploidy, meaning that samples are considered to have amplification of the gene if ASCATploidy is < 2.7 and the gene copy number is > 5 or where ASCATpoidy is >=2.7 and the gene copy number is >=9. b) Boxplots of beta values for samples with complex chromosome 8 (n = 8, red) and others (n = 61, blue). c) Boxplots of beta values for samples with kataegic events at chromosome 8 (n = 18, red) and others (n = 51, blue). All box plots show the median values with the interquartile range (lower and upper hinge) and ± 1.5-fold the interquartile range from the first and third quartile (lower and upper whiskers), extreme outliers are shown as asterisks ‘*’. Source data are provided as a Source Data file.

_
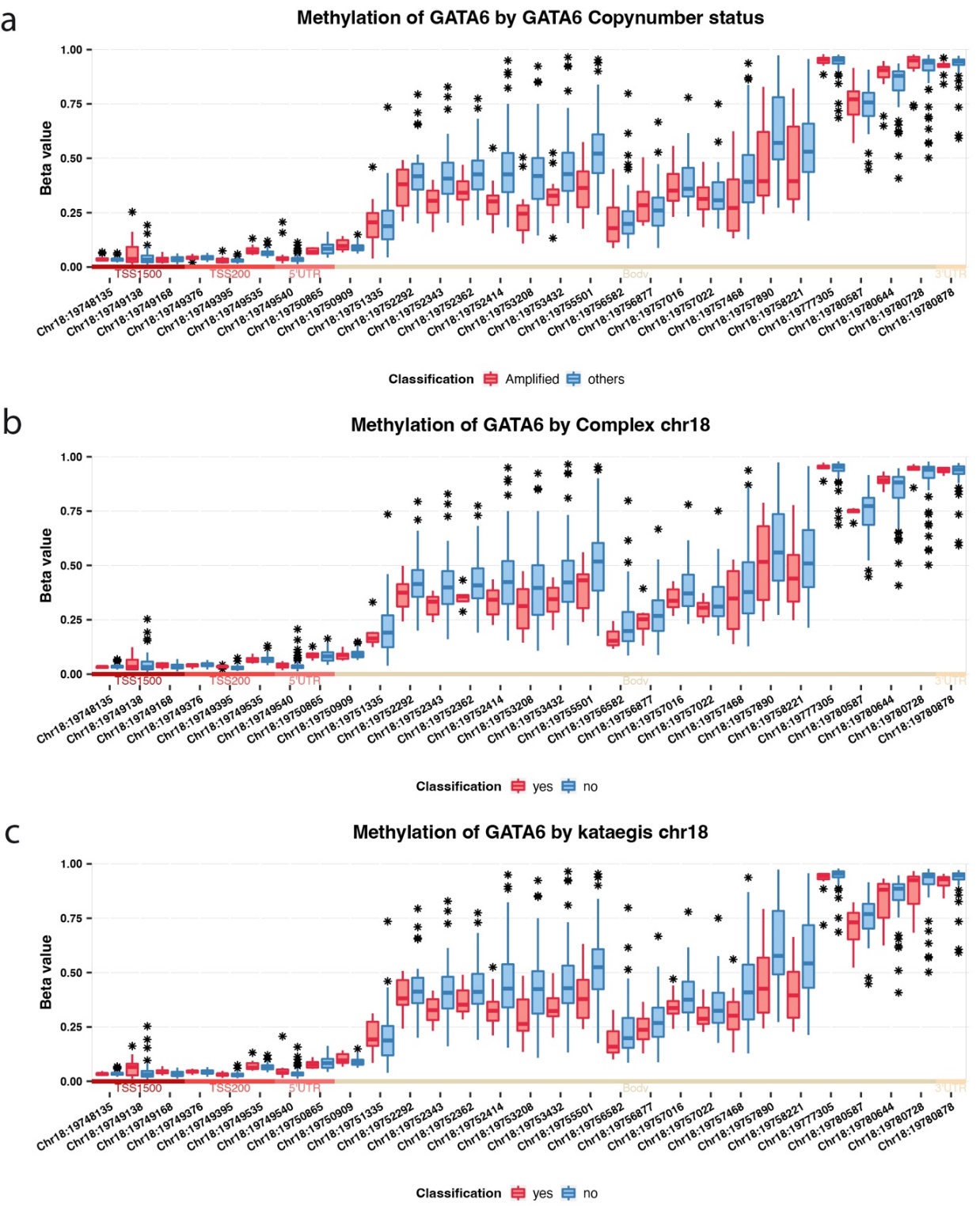
_

**Supplementary Figure 13: Methylation status of *GATA6* promoter and gene-body**. Data in this figure is derived from n = 69 biologically independent samples. a) Boxplots of beta values in samples with amplified *GATA6* copy number status (n = 12, red) and others (n = 57, blue). Amplified status of the gene of interest has been corrected for ploidy, meaning that samples are considered to have amplification of the gene if ASCATploidy is < 2.7 and the gene copy number is > 5 or where ASCATpoidy is >=2.7 and the gene copy number is >=9. b) Boxplots of beta values for samples with complex chromosome 18 (n = 7, red) and others (n = 62, blue). c) Boxplots of beta values for samples with kataegic events at chromosome 18 (n = 14, red) and others (n = 55, blue). All box plots show the median values with the interquartile range (lower and upper hinge) and ± 1.5-fold the interquartile range from the first and third quartile (lower and upper whiskers), extreme outliers are shown as asterisks ‘*’. Source data are provided as a Source Data file.

**
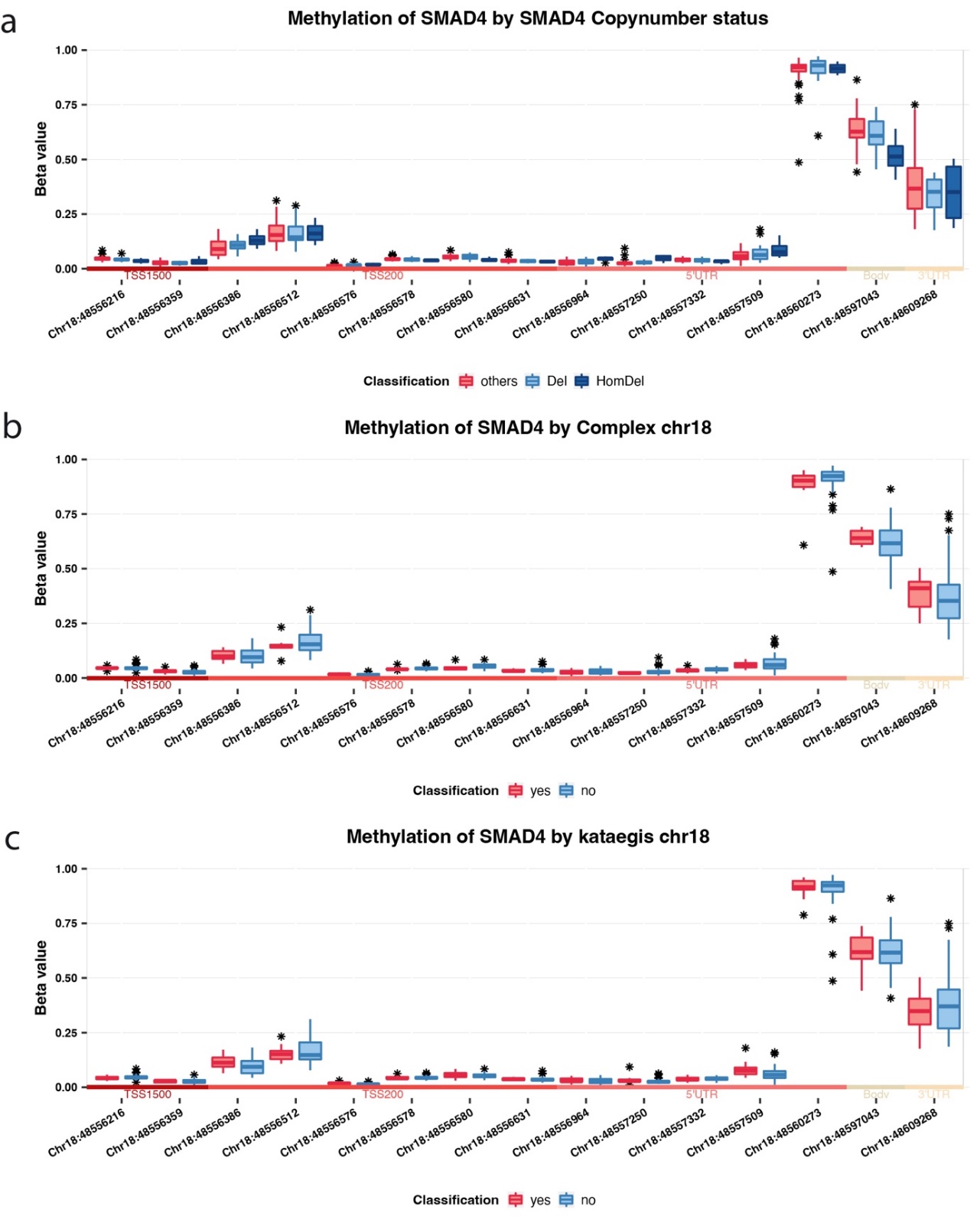
**

**Supplementary Figure 14: Methylation status of *SMAD4* promoter and gene-body**. Data in this figure is derived from n = 69 biologically independent samples. a) Boxplots of beta values in samples with homozygous deletion (n = 4, HomDel, CN0, dark blue), Deletion (n = 19, Del, CN1, blue) *SMAD4* copy number status and others (n=46, red). b) Boxplots of beta values for samples with complex chromosome 18 (n = 7, red) and others (n = 62, blue). c) Boxplots of beta values for samples with kataegic events at chromosome 18 (n = 14, red) and others (n = 55, blue). All box plots show the median values with the interquartile range (lower and upper hinge) and ± 1.5-fold the interquartile range from the first and third quartile (lower and upper whiskers), extreme outliers are shown as asterisks ‘*’. Source data are provided as a Source Data file.

_­_­­
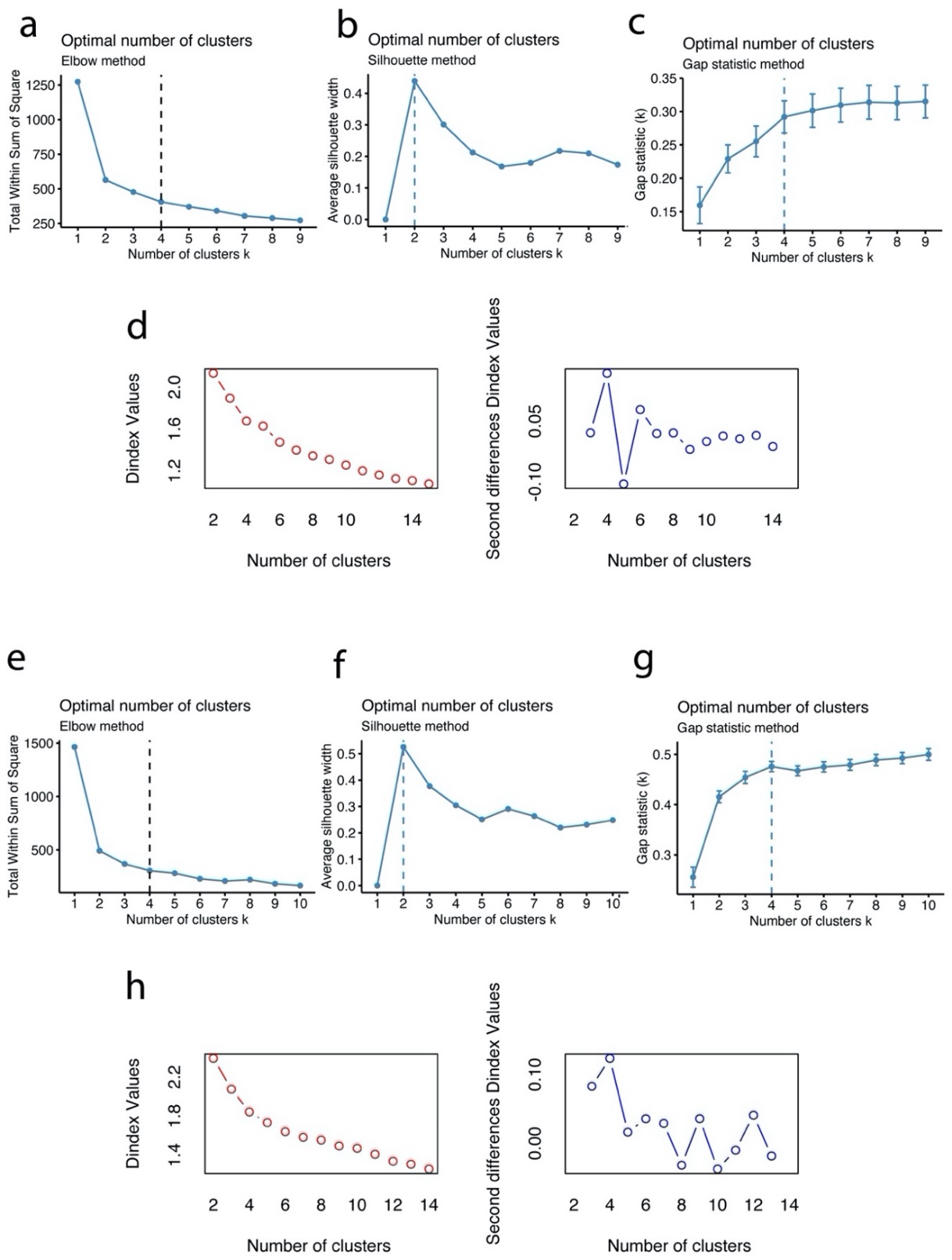


**Supplementary Figure 15: Optimal number of clusters for k-mean clustering in our study cohort and TCGA cohort.** The optimal number of clusters for unsupervised clustering in our ­study cohort (n = 68 biologically independent samples with RNA-seq) according to: a) Elbow method; b)**­** Silhouette method; c) Gap statistic method (with errors bars as mean values +/- SEM), and d) Euclidean distance for k-mean clustering. The optimal number of clusters for unsupervised clustering of TCGA cohort (n = 78 biologically independent samples with RNA-seq) according to: e) Elbow method; f)**­** Silhouette method; g) Gap statistic method (with errors bars as mean values +/- SEM), and h) Euclidean distance for k-mean clustering. Three out of four methods suggest four clusters as the optimal number to analyse our and TCGA cohorts. Source data used to calculate values are provided as a Source Data file.


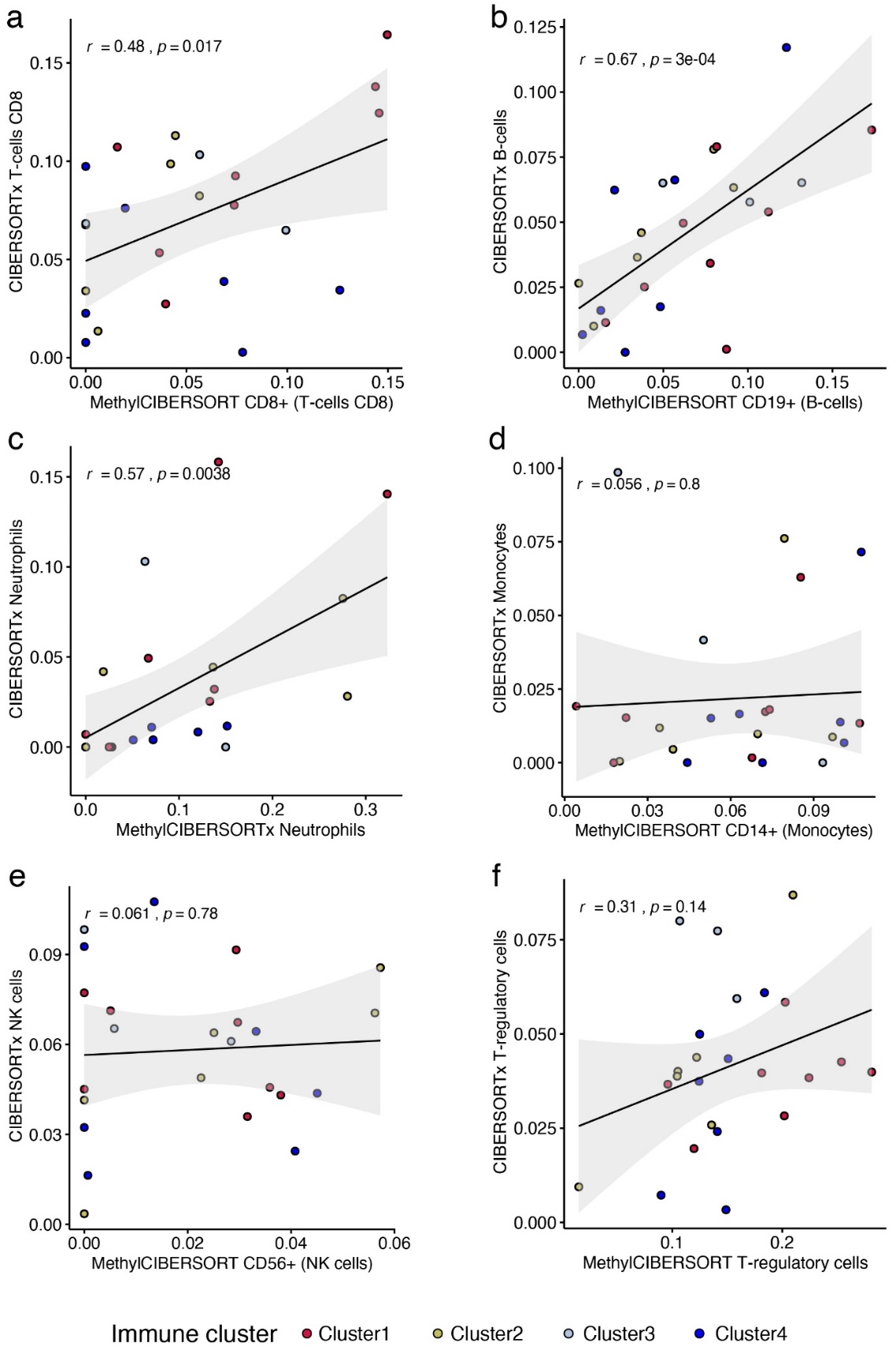


**Supplementary Figure 16: Pearson correlation between CIBERSORTx and MethylCIBERSORT.** Pearson correlation (two-sided) to compare the proportion of cell types estimated by RNA-seq using CIBERSORTx (y-axis) and methylation profiling using MethylCIBERSORT (x-axis) for tumours with RNA-seq and methylation data (n = 24 biologically independent tumour samples). The correlation of six cell types were assessed: a) T-cell CD8; b) B-cells; c) Neutrophils; d) Monocytes; e) NK cells; f) T-regulatory cells. In all plots, samples are colour coded according to their immune cluster with cluster 1 (n = 8, red), cluster 2 (n = 6, yellow), cluster 3 (n = 3, light blue) and cluster 4 (n = 7, blue). Shading indicates 95% confidence intervals. Source data are provided as a Source Data file.

_­
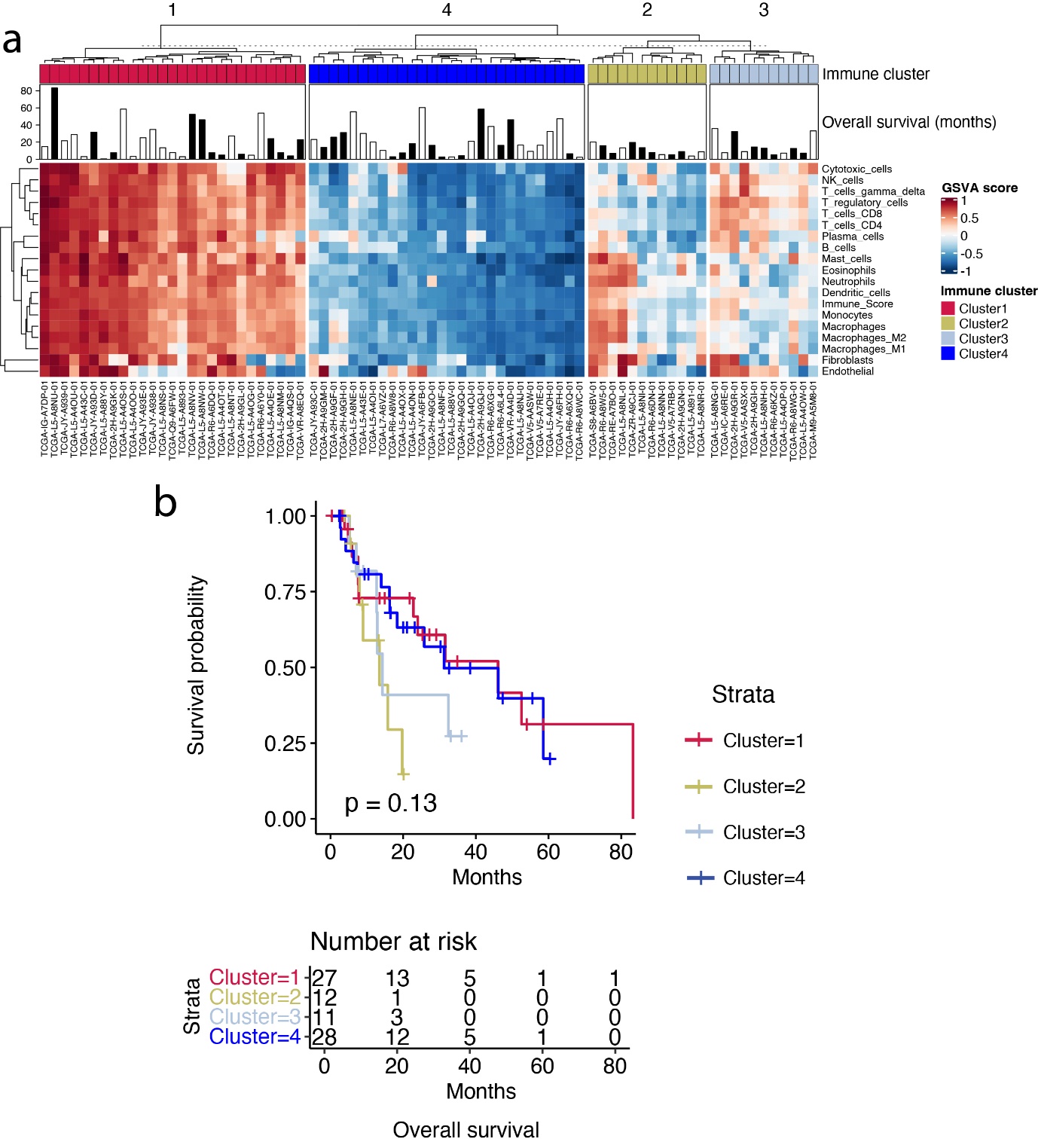
­­_

**Supplementary Figure 17: Immune microenvironment of TCGA cohort.** a) Unsupervised k-mean clustering of 78 TCGA ESCA samples which were classified as oesophageal adenocarcinoma using GSVA scores from ConsensusTME transcriptomic deconvolution of 18 immune cell types. The colour bar above the figure represents the immune cell clusters which are consistent with immune subtypes in our study cohort. The histogram is the overall patient survival, white bars represent patients who are alive and black who are dead. b) Kaplan-Meier plot (log-rank test) comparing overall survival of TCGA immune subtype with log-rank test comparing the four immune subtypes. The number of patients in each group is indicated below the plots. Source data are provided as a Source Data file.


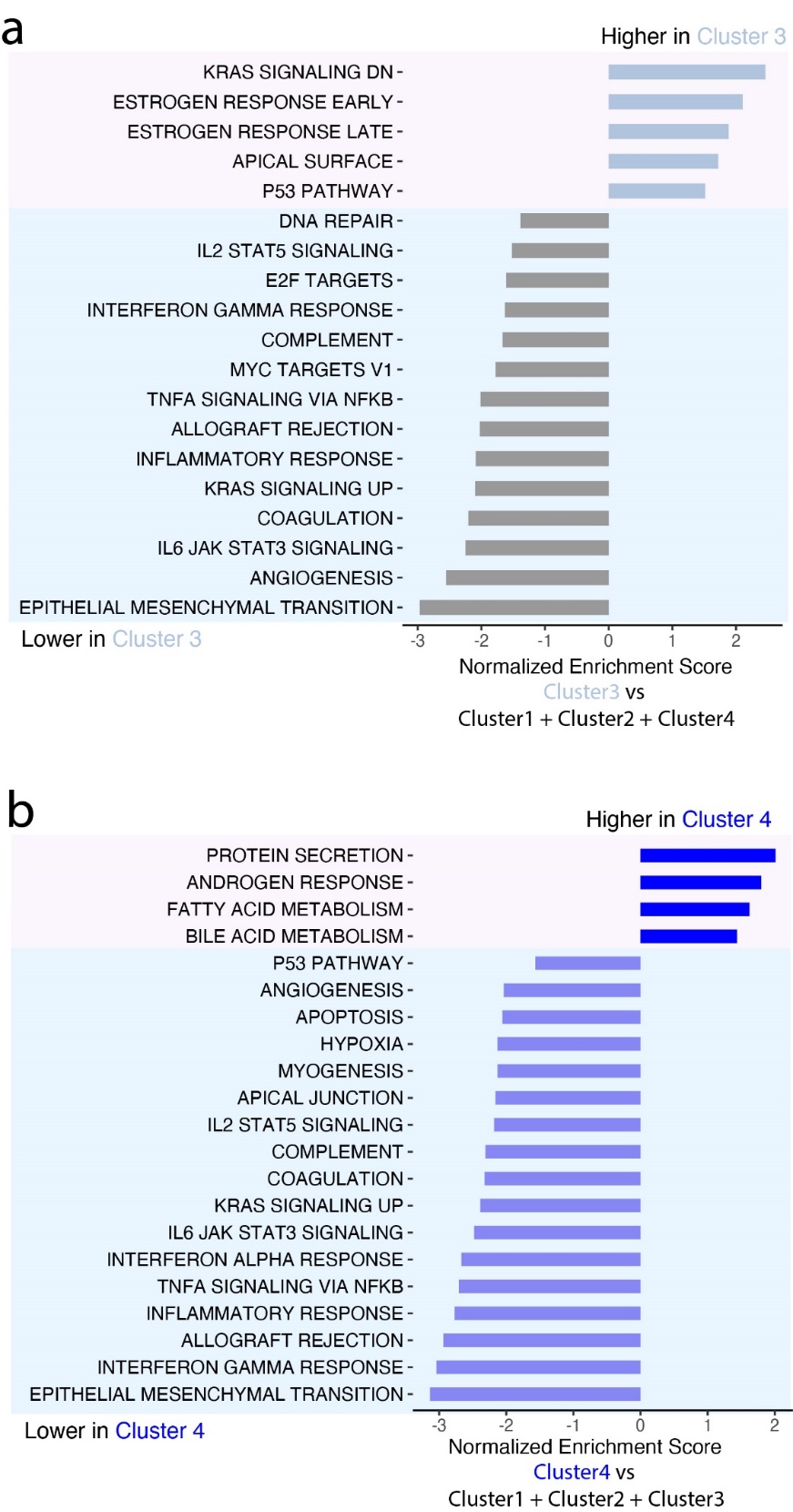


**Supplementary Figure 18: Gene set enrichment analysis of Cluster 3 and Cluster 4.** a) Bar plot representing normalised enrichment scores (x-axis) and enriched pathways (y-axis) in samples within Cluster 3 compared to other clusters. Represented pathways had an adjusted p-value < 0.05 with GSEA, the pink background represents up-regulated pathways and the blue background shows down-regulated pathways. The represented pathways had an adjusted p-value < 0.05. b) Bar plot representing normalised enrichment scores (x-axis) and enriched pathways (y-axis) in samples within Cluster 4 compared to other clusters. Represented pathways had an adjusted p-value < 0.05 with GSEA, the, pink background represents up-regulated pathways and the blue background shows down-regulated pathways. Source data are provided as a Source Data file.


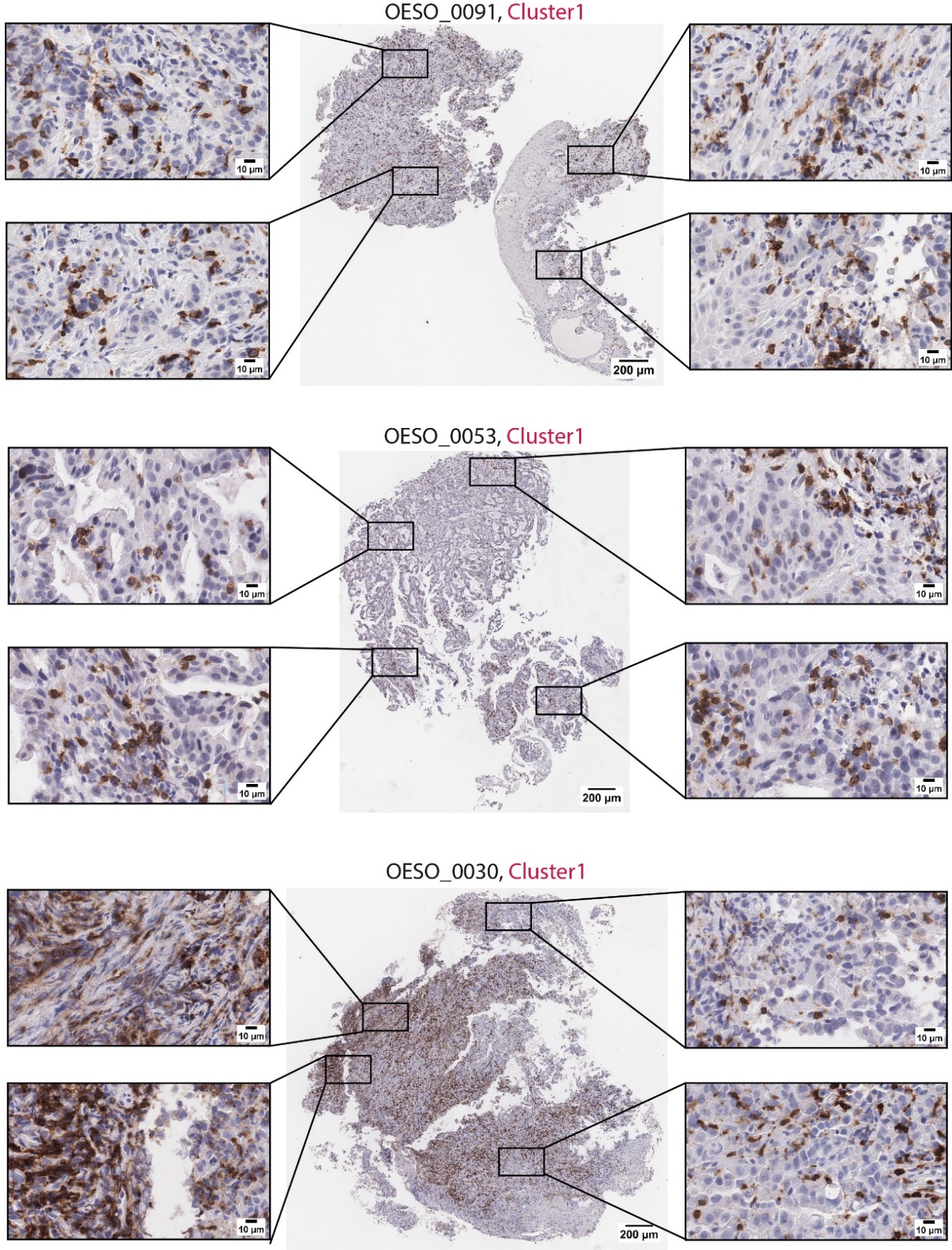


**Supplementary Figure 19: Heterogeneity of CD8 immunohistochemistry in tumours with an immune hot microenvironment (Cluster 1).** Whole slide and zoomed-in images from different tumour regions showing intra-tumour heterogeneity of CD8 T-cell staining within the immune hot microenvironment. The boxes indicate the approximate position of each zoomed image. Images scanned at 40x magnification, scale bar shown on image. Images shown for three Cluster 1 samples (from n = 13 Cluster 1 samples with IHC) from top to bottom are: OESO_0091, OESO_0053 and OESO_0030.

**
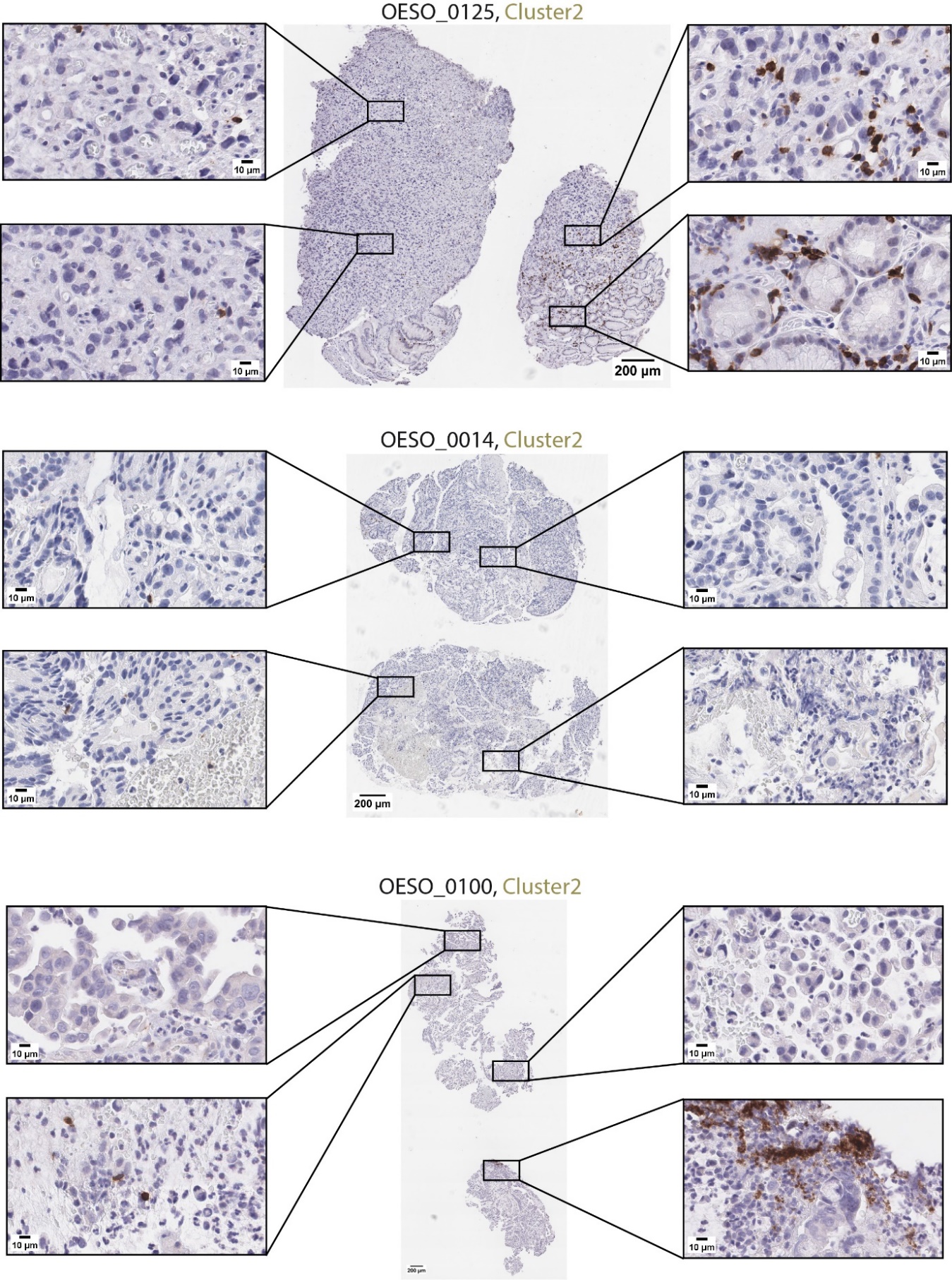
**

**Supplementary Figure 20: Heterogeneity of CD8 immunohistochemistry in tumours with an immune suppressed microenvironment (Cluster 2).** Whole slide and zoomed-in images from different tumour regions showing intra-tumour heterogeneity of CD8 T-cell staining within the microenvironment. The boxes indicate the approximate position of each zoomed image. Images scanned at 40x magnification, scale bar shown on image. Images shown for three Cluster 2 samples (from n = 6 Cluster 2 samples with IHC) from top to bottom are: OESO_0125, OESO_0014 and OESO_0100.

_
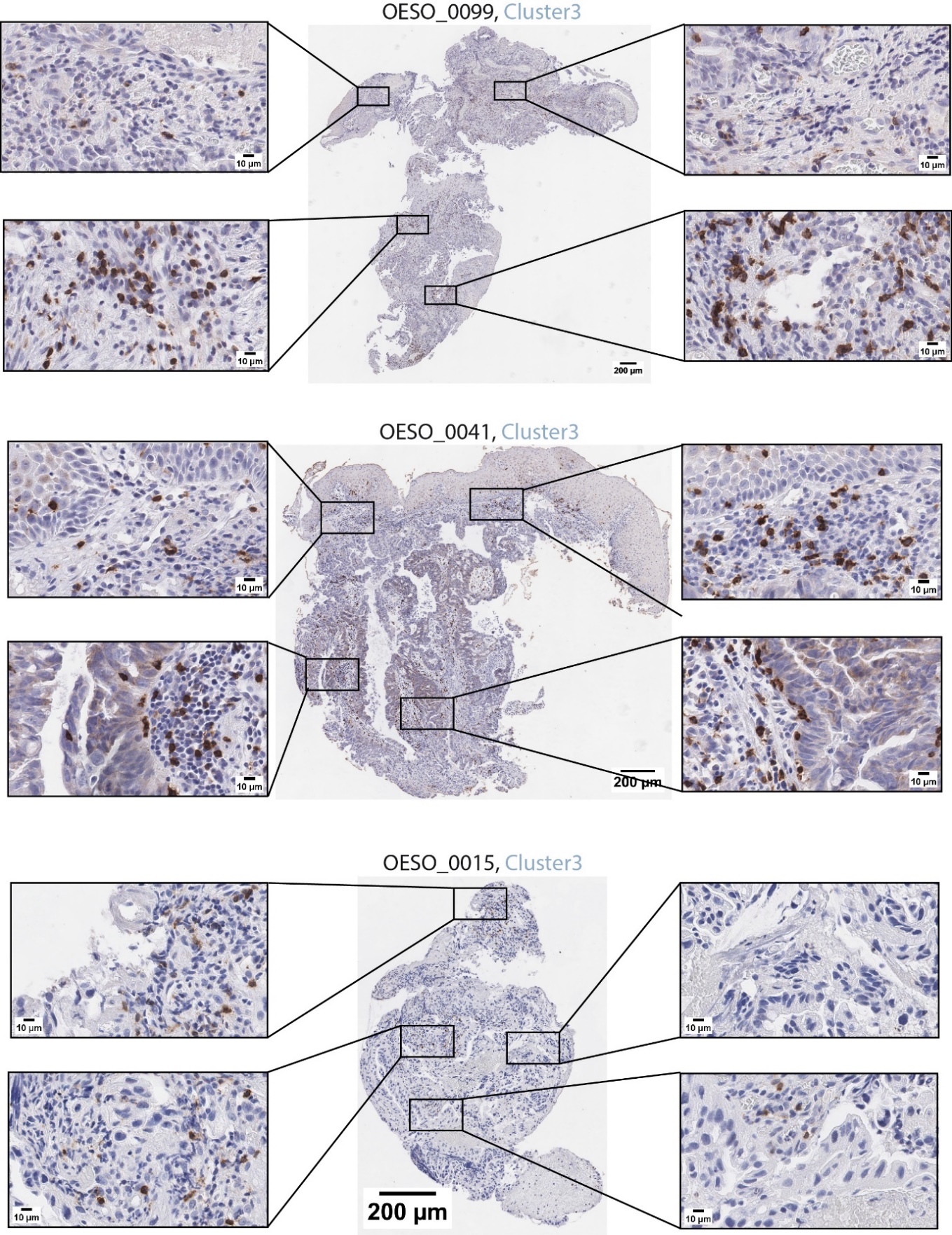
_

**Supplementary Figure 21: Heterogeneity of CD8 immunohistochemistry in tumours with an immune moderate microenvironment (Cluster 3).** Whole slide and zoomed-in images from different tumour regions showing intra-tumour heterogeneity of CD8 T-cell staining within the microenvironment. The boxes indicate the approximate position of each zoomed image. Images scanned at 40x magnification, scale bar shown on image. Images shown for all three Cluster 3 samples with IHC, from top to bottom are: OESO_0099, OESO_0041 and OESO_0015.

**
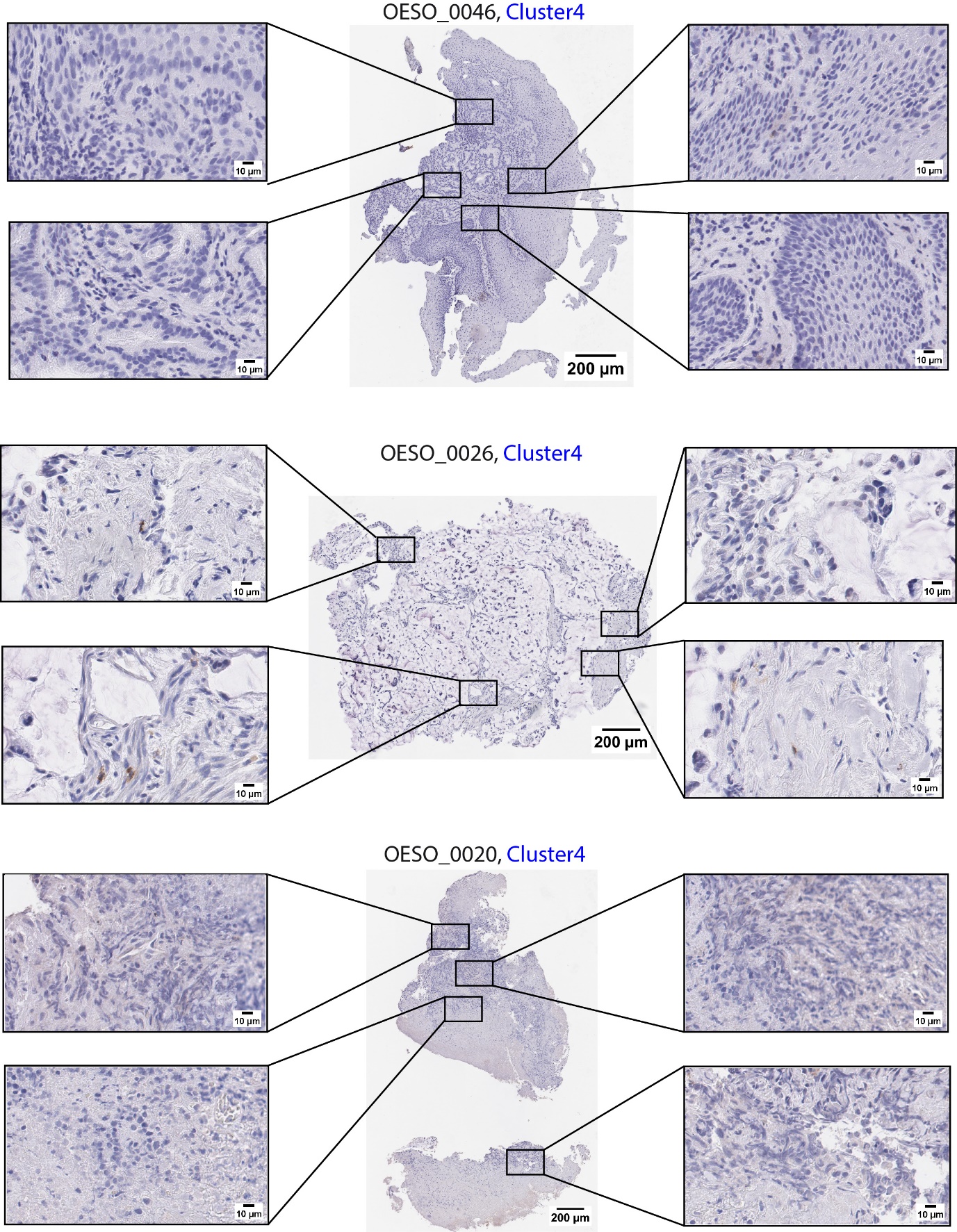
**

**Supplementary Figure 22: Heterogeneity of CD8 immunohistochemistry in tumours with an immune cold microenvironment (Cluster 4).** Whole slide and zoomed-in images from different tumour regions showing a lack of CD8 T-cell staining within the microenvironment. The boxes indicate the approximate position of each zoomed image. Images scanned at 40x magnification, scale bar shown on image. Images shown for three Cluster 4 samples (from n = 10 Cluster 4 samples with IHC) from top to bottom are: OESO_0046, OESO_0026 and OESO_0020.

**
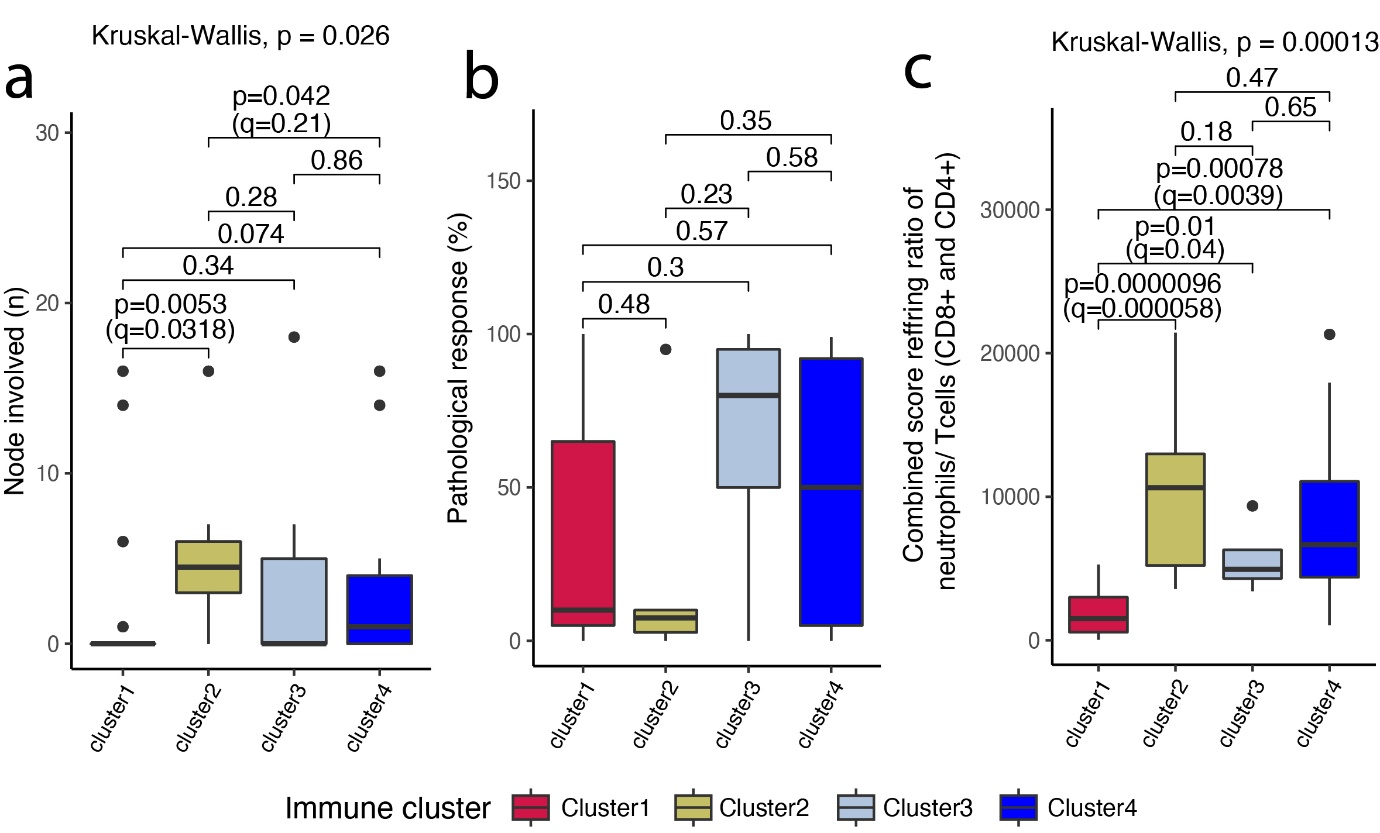
**

**Supplementary Figure 23: Clinico-pathological features of samples in each immune cluster.** Data for samples with RNA-seq (n = 68 biologically independent samples) stratified by immune cluster (Cluster 1 n = 24, Cluster 2 n = 12, Cluster 3 n = 12 and Cluster 4 n = 20). a) Box plot showing the number of nodes involved within tumours of each immune cluster type (Wilcoxon rank sum two-sided test). b) The percentage of pathological response within tumours of each immune clusters (Wilcoxon rank sum two-sided test). c) A combined ranking score representing the ratio of neutrophils to T-cells (CD4 + CD8) in tumours within each immune cluster (Wilcoxon rank sum two-sided test). Box plots in a, b and c show the median values with the interquartile range (lower and upper hinge) and ± 1.5-fold the interquartile range from the first and third quartile (lower and upper whiskers). p-values from Wilcoxon rank sum test (two-sided) is used for paired analysis. Kruskal-Wallis is used for multiple groups comparison. The ratio of neutrophils to T-cells was estimated using GSVA scores of neutrophils, CD4+ T-cells and CD8+ T-cells. As we are not able to calculate the ratio directly from GSVA scores as the ranges are -1 to 1, we first ranked the GSVA score from “high to low” to infer positive correlation of neutrophils ranking score, and “low to high” to infer negative correlation of T-cells (CD4+ and CD8+) ranking scores. Meaning that *rank_m_ = GSVA score for neutrophils -> ranking from high to low; rank_cd4_ = GSVA score for CD4+ T-cells -> ranking from low to high; rank_cd8_ = GSVA score for CD4+ T-cells -> ranking from low to high.* A combined ranking score was estimated to infer the ratio of neutrophils to CD4+ T-cells and CD8+ T-cells as follows: *Combined ranking score= rank_m_ × rank_cd4_ × rank_cd8_*. Source data are provided as a Source Data file.

_­_


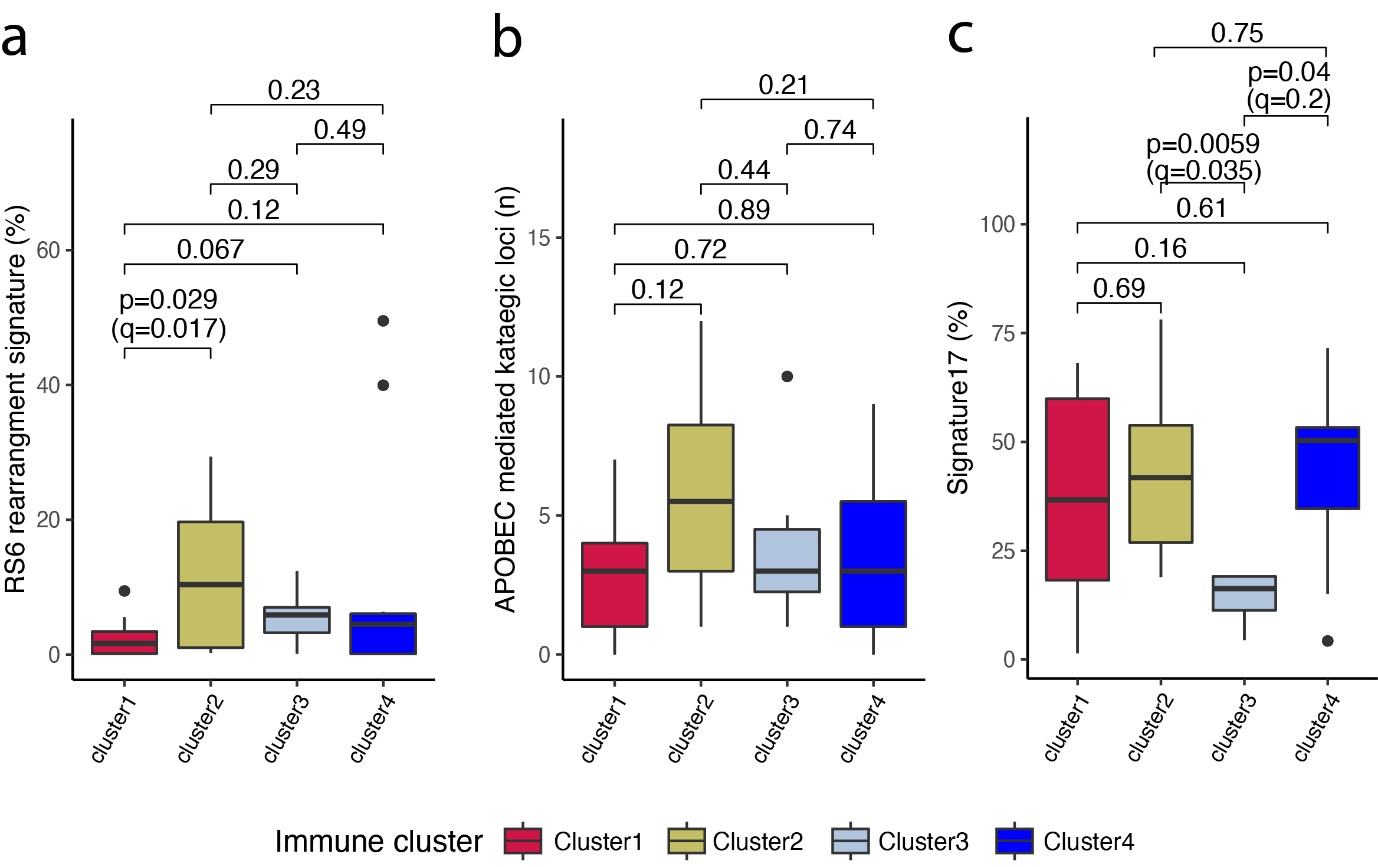


**Supplementary Figure 24: Genomic features associated with the immune clusters.** Tumours with both WGS and RNA sequencing (n = 45 biologically independent samples) were included in the analysis, with samples stratified by immune cluster (Cluster 1 n = 14, Cluster 2 n = 11, Cluster 3 n = 7 and Cluster 4 n = 13). a) Box plot showing the percent of RS6 rearrangement signature in samples within the four immune clusters (Wilcoxon rank sum two-sided test). b) Box plot showing the number of APOBEC-mediated kataegic loci in samples within the four immune clusters (Wilcoxon rank sum two-sided test). c) Box plot showing the percent of Signature 17 in samples within the four immune clusters (Wilcoxon rank sum two-sided test). Box plots in a, b and c show the median values with the interquartile range (lower and upper hinge) and ± 1.5-fold the interquartile range from the first and third quartile (lower and upper whiskers). Source data are provided as a Source Data file.


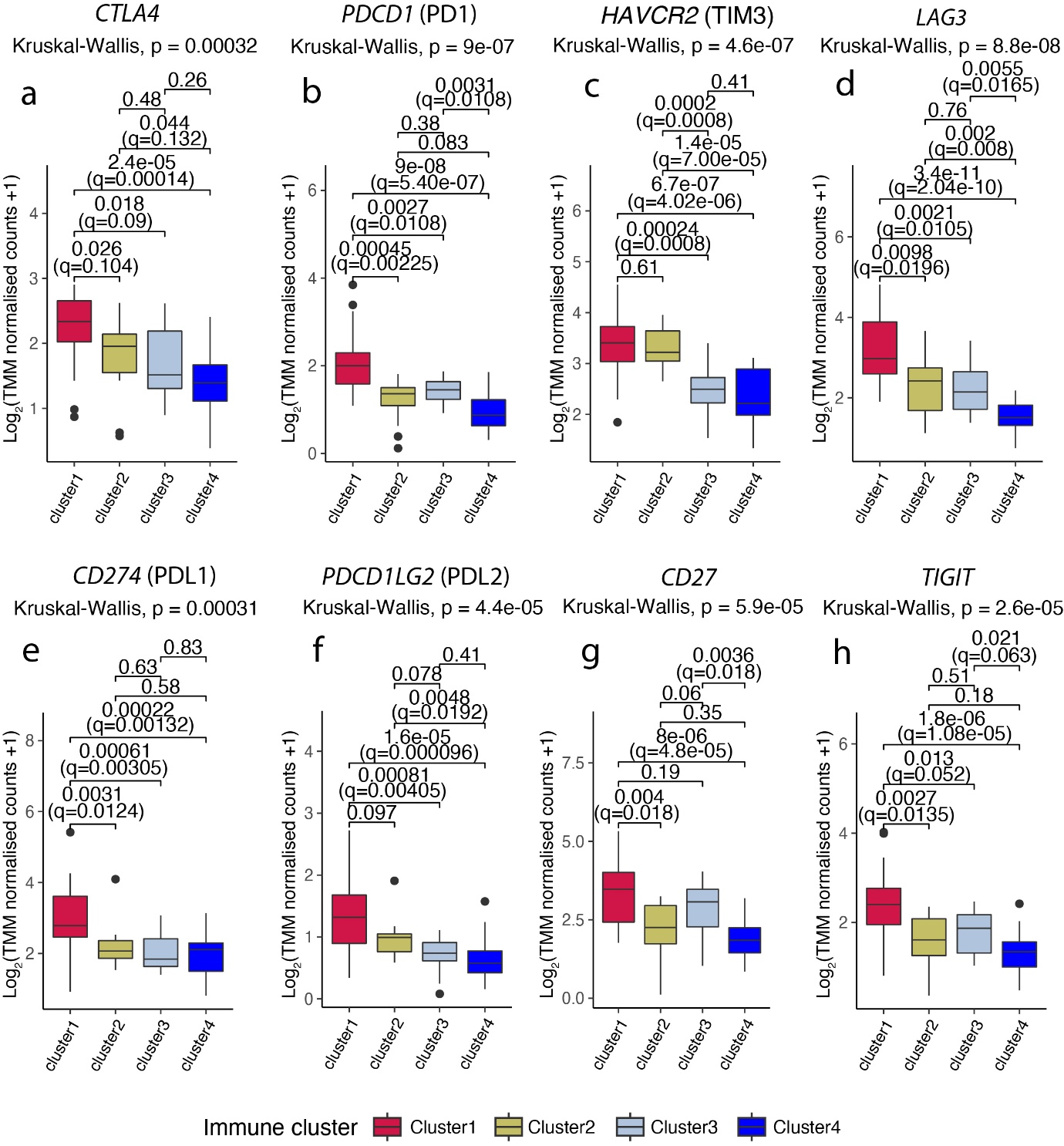


**Supplementary Figure 25: Immune checkpoint molecules associated with the immune clusters.** Log scale TMM normalised RNA expression of samples with RNA-seq were included in analysis (n = 68 biologically independent samples), with samples stratified by immune cluster (Cluster 1 n = 14, Cluster 2 n = 11, Cluster 3 n = 7 and Cluster 4 n = 13). Box plots show the TMM normalised RNA expression for samples in each immune cluster for the following genes (with corresponding protein name in brackets): a) *CTLA4*, b) *PDCD1* (PD1), c) *HAVCR2* (TIM3), d) *LAG3*, e) *CD274* (PDL1), f) *PDCD1LG2* (PDL2), g) *CD27* and h) *TIGIT*. Box plots in panels a-h show the median values with the interquartile range (lower and upper hinge) and ± 1.5-fold the interquartile range from the first and third quartile (lower and upper whiskers). p-values from Wilcoxon rank sum two-sided test is used for paired analysis. Kruskal-Wallis is used for multiple groups comparison. Source data are provided as a Source Data file.
