# Supplementary material for: Multi-omic features of oesophageal adenocarcinoma in patients treated with preoperative neoadjuvant therapy
Source: Nat Commun. 2023 May 31;14:3155. doi: 10.1038/s41467-023-38891-x (PMC10232490; doi:10.1038/s41467-023-38891-x)
Supplement: Supplementary file 3 — Description to Additional Supplementary Information [file 41467_2023_38891_MOESM3_ESM.pdf]

Description of Additional Supplementary Files

Supplementary Data 1: Clinical information of oesophageal adenocarcinoma patients and data availability

Supplementary Data 2: Whole genome sequencing details and identified genomic features of oesophageal adenocarcinoma samples

Supplementary Data 3: Somatic SNVs and INDEL variants identified in this cohort

Supplementary Data 4: Combined percent of APOBEC mediated complex events

Supplementary Data 5: Cell type estimations and identified immune clusters
